# Hybrid Semi‐Transparent Perovskite‐Photovoltaics and Concentrated Solar‐Thermal Harvesting Collectors

**DOI:** 10.1002/advs.78362

**Published:** 2026-10-20

**Authors:** Nada. A. Alati, Parth H. Arya, Yu Tian, Ashok Reddy, David B. Ritzer, Bahram Abdollahi Nejand, Ulrich W. Paetzold, Bryce S. Richards, Gan Huang

**Affiliations:** ^1^ Institute of Microstructure Technology Karlsruhe Institute of Technology Eggenstein‐Leopoldshafen Germany; ^2^ Light Technology Institute Karlsruhe Institute of Technology Karlsruhe Germany

**Keywords:** perovskite, photovoltaic thermal, photovoltaics, solar cell, solar energy, thermal energy

## Abstract

Photovoltaics and solar thermal collectors each face limitations in efficiently harnessing solar energy. This study presents a hybrid semi‐transparent perovskite‐photovoltaic and concentrating solar thermal (SPV‐CST) collector designed to maximize solar energy utilization through spectral splitting. The perovskite solar cell captures both diffuse and direct solar irradiance, converting shorter‐wavelength sunlight into electricity while transmitting the near‐infrared spectrum. The SPV‐CST collector achieves effective thermal decoupling between the photovoltaic and thermal components. Specifically, the lens concentrates the transmitted light onto a solar‐thermal collector, producing heat at temperatures ranging from 88.0°C to 154.9°C. Meanwhile, the operating temperature of the perovskite solar cell is limited to 38.5°C to reduce thermal stability issues and preserve its electrical efficiency at 13.8%. Using state‐of‐the‐art semi‐transparent perovskite cells from the literature, the SPV‐CST collector is projected to improve its electrical efficiency from 13.8% to 21.3%, with only a slight reduction in thermal efficiency from 20.7% to 19.2% at 100°C output temperature. This work provides insights into the design, modeling, and operational optimization of hybrid solar collectors, paving the way for sustainable solar energy applications across diverse conditions and climates.

## Graphical abstract

A hybrid semi‐transparent perovskite‐photovoltaic and concentrating solar‐thermal collector is developed to spectrally split sunlight for simultaneous electricity and heat generation. The design achieves thermal decoupling between the photovoltaic and thermal components, offering a promising route for efficient co‐harvesting of solar electricity and high‐temperature heat from a single collector.

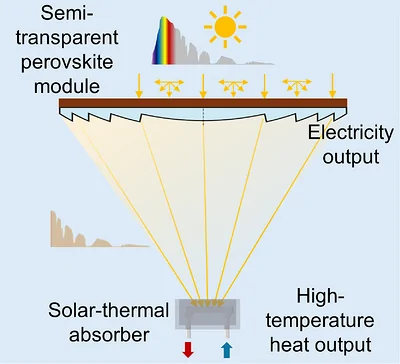

## 1 Introduction

Solar energy is the world's most abundant, green, and renewable energy source, holding immense promise for the transition to cleaner energy systems. Photovoltaic (PV) and solar thermal technologies are the primary methods for harnessing solar radiation. While the solar thermal sector continues to expand, its overall growth has slowed in recent years. The global cumulative installed capacity of solar thermal collectors reached approximately 544 GW_th_ at the end of 2024, following 17.8 GW_th_ of new installations, representing a decline of about 14% compared with 2023 [1]. In contrast, the PV sector has sustained a strong upward trend, with global cumulative PV capacity rising to roughly 2,156 GW_p_ by the end of 2024, corresponding to a compound annual growth rate of about 27% between 2014 and 2024 [2]. In terms of energy output, solar thermal systems supplied approximately 443 TWh of thermal energy in 2024 [1], while photovoltaics generated around 1641 TWh of electricity in 2024 [2]. Heating accounts for a significant share of global energy demand, representing nearly half of total energy consumption across the residential and industrial sectors. Thus, advancing solar energy systems for both electricity and heat production is critical for a successful transition to sustainable energy solutions. At the same time, increasing population and rising energy demand place growing pressure on the limited surface areas available for solar deployment, particularly rooftops and building facades. To maximize energy yield per unit area, higher solar conversion efficiencies are essential.

PV is limited in its ability to harness the full solar spectrum, as it can only utilize photons with energies exceeding the semiconductor bandgap. Several loss mechanisms hinder the efficiency of solar cells. For photons with energies below the bandgap, transparency losses occur as these photons pass through the material without being absorbed. For photons with energies significantly above the bandgap, thermalization losses dominate, where excess energy is dissipated as heat, raising the PV cell's operating temperature and further reducing its efficiency.

Hybrid PV‐thermal collectors offer a potential solution for more efficient solar energy utilization by co‐generating electricity and heat. In these systems, a thermal absorber is coupled with the PV cells to extract waste heat, enhancing electrical efficiency while producing useful thermal energy [3]. However, conventional PV‐thermal collectors face a trade‐off between electrical and thermal performance. To prioritize electricity generation, the thermal absorber is typically maintained at temperatures below 60°C to prevent degradation of the PV cells' electrical efficiency [4]. This limitation highlights the importance of thermal decoupling between the PV cells and the thermal absorber to further optimize hybrid systems.

Spectral splitting has emerged as a promising approach to achieve thermal decoupling and enable more efficient utilization of the solar spectrum [5, 6, 7, 8]. By selectively harvesting specific parts of the spectrum, this technique can simultaneously optimize electricity and heat generation. Spectral splitting can be achieved using optical filters, such as film‐based or liquid‐based filters [9, 10, 11]. Thin‐film spectral splitters typically employ multilayer dielectric or nano‐structured optical coatings that selectively transmit the wavelength range suitable for photovoltaic conversion while reflecting or redirecting the remaining spectrum for thermal harvesting [12, 13]. Recent studies continue to demonstrate high spectral selectivity using multilayer beam‐splitting films in PV‐thermal collectors. Liquid‐based spectral splitters generally employ wavelength‐selective absorbing fluids, particularly nanofluids containing metallic, metal‐oxide, carbon‐based, or core‐shell nanoparticles [14, 15, 16]. Their optical response can be tailored through nanoparticle composition, size, concentration, and optical path length, while the fluid can simultaneously serve as a heat‐transfer medium. Recent reviews and system‐level studies have highlighted the rapid development of nanofluid‐based spectral‐splitting PV‐thermal collectors and their potential for thermally decoupled full‐spectrum solar‐energy utilization [17].

However, these filters introduce challenges, including optical losses that reduce PV efficiency and increased system complexity [18]. An alternative method involves using semi‐transparent solar cells as an optical filter. Such PV devices selectively absorb part of the solar spectrum for electricity generation while transmitting the remainder to a thermal receiver for heat production. The experimental work by Codd et al. [19], Liang et al. [20]. and Huang et al. [21], and the modeling study by Huang and Markides [22] have demonstrated the feasibility of integrating semi‐transparent PV cells with solar thermal absorbers under concentrated sunlight to achieve high‐temperature heat output. They also noted challenges in managing PV cell temperatures under high solar concentration.

Recently, perovskite PV cells have garnered significant attention. Perovskite PV cells have already demonstrated high power conversion efficiencies [23], though further efforts are needed to improve their stability [24] and scalability [25]. Perovskite solar cells offer a tuneable bandgap ranging from 1.5 to 2.3 eV by varying their chemical composition, which is higher than the indirect bandgap of conventional silicon cells (1.12 eV). This allows a larger portion of the solar spectrum, specifically photons with wavelengths in the near‐infrared (e.g., > 775 nm for a bandgap of 1.6 eV), to pass through the semi‐transparent perovskite PV cells for heat generation, making them highly promising for next‐generation spectral‐splitting hybrid PV‐thermal collectors. However, the design and performance of these emerging solar collectors using perovskite solar cells remain unclear.

In this study, we present a hybrid semi‐transparent perovskite‐photovoltaic and concentrating solar‐thermal (SPV‐CST) collector that employs spectral splitting to simultaneously generate electricity and high‐temperature heat. The system integrates a semi‐transparent perovskite (SPV) module with a bandgap of 1.6 eV (corresponding to 775 nm) to split the solar spectrum and generate electricity. The SPV module is positioned above a Fresnel lens to capture both diffuse and direct sunlight (< 775 nm) before the sunlight being concentrated by the Fresnel lens, eliminating the need for intensive active cooling for the SPV module. The Fresnel lens then concentrates transmitted sunlight (> 775 nm) onto a solar thermal absorber to produce high‐temperature heat. This configuration allows the solar thermal receiver to achieve high output temperatures without overheating the SPV module, offering an efficient approach to hybrid solar energy harvesting. To achieve normal incidence of sunlight on the Fresnel lens in a practical system, a biaxial tracker is required. In this study, however, the focus is on modeling and proof‐of‐concept testing under a solar simulator; therefore, a solar tracker is not employed in this research.

## 2 Results

### 2.1 The Concept of Hybrid Semi‐Transparent Perovskite‐Photovoltaics and Concentrated Solar‐Thermal (SPV‐CST) Collectors

The design concept of the SPV‐CST solar collector is illustrated in Figure 1. A SPV module, with an approximate area of 1 m^2^, is positioned above a Fresnel lens to capture both direct and diffuse solar irradiance. The SPV module selectively absorbs photons with energy equal to or greater than its bandgap (i.e., > 1.6 eV, or corresponding to < 775 nm), converting them into electricity. Photons that cannot be utilized for electricity generation (> 775 nm) are transmitted through the SPV module. These transmitted photons are then concentrated by the Fresnel lens onto a concentrated solar thermal (CST) absorber, which generates high‐temperature thermal energy.

**FIGURE 1:**
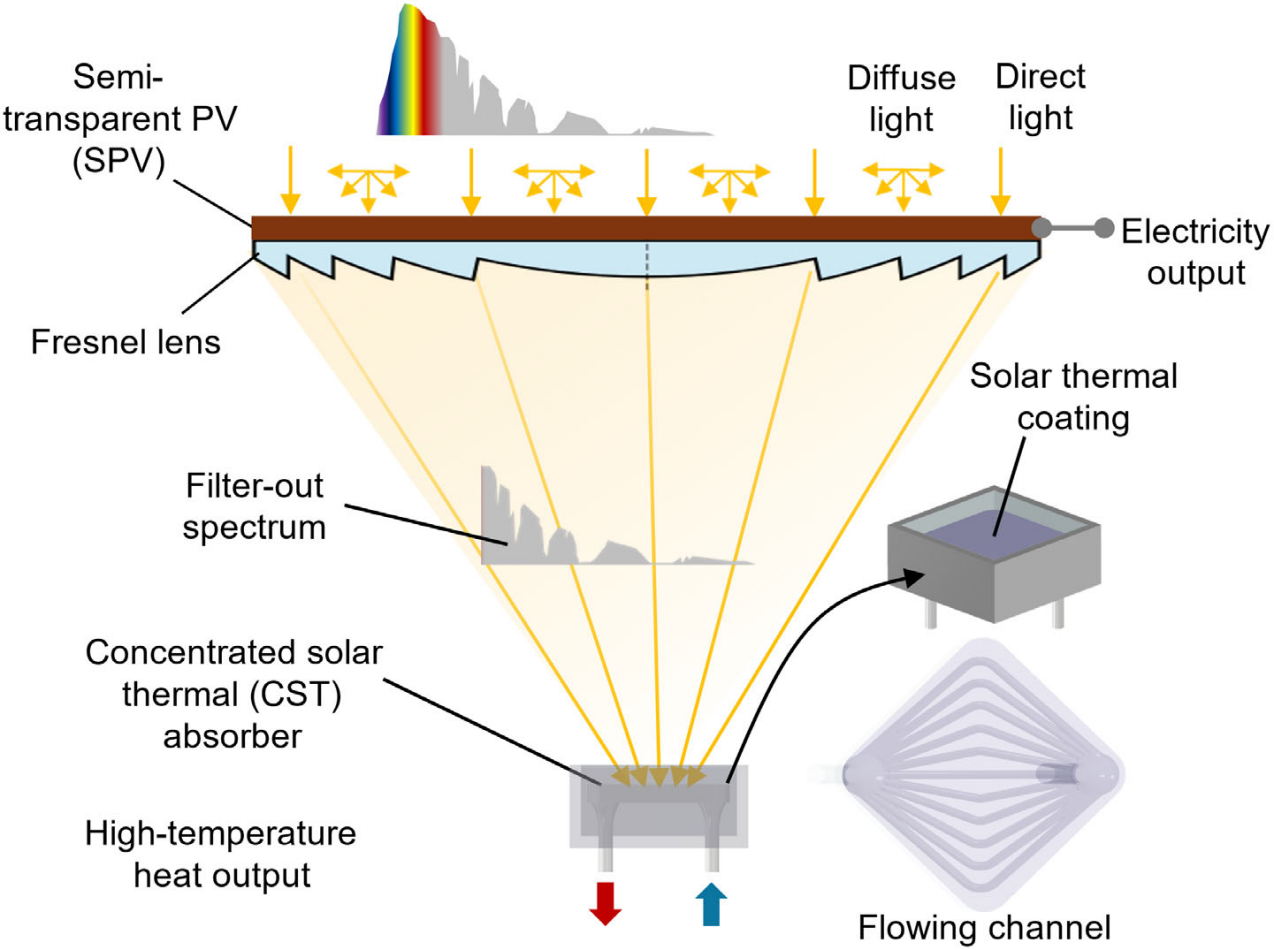
Schematic diagram illustrating the design concept of the hybrid semi‐transparent perovskite‐photovoltaic and concentrated solar thermal (SPV‐CST) collector. The SPV module is positioned above a Fresnel lens, absorbing a portion of the global solar irradiance to generate electricity. The Fresnel lens concentrates only the transmitted direct solar irradiance onto the solar thermal absorber. For example, the direct and diffuse fractions in AM1.5G (Air Mass 1.5 Global Spectrum) are ∼90% and ∼10%.

It is important to note that natural sunlight contains a significant proportion of diffuse light. For instance, approximately 21% and 51% of incident sunlight is diffuse in Cairo and Berlin, respectively [26]. In conventional concentrated PV‐thermal collectors, only direct sunlight can be concentrated and utilized, leaving all the diffuse light unexploited. However, in this SPV‐CST design, the diffuse light can be fully utilized for electricity generation, with only the diffuse near‐infrared component being unused for heat production.

The CST absorber is coated with a spectrally‐selective coating that has a high absorption for the solar spectrum range and a low thermal emissivity in the middle infrared range to maximize solar absorption and reduce radiative heat loss. The built‐in channels are incorporated within the CST absorber to efficiently transfer heat to the heat transfer fluid. The CST absorber is housed inside a vacuum chamber to reduce heat convection loss. The inner walls of the chamber are provided with a low‐thermal‐emissivity coating to minimize thermal radiation loss. In this design, the CST absorber is placed on the focus of the Fresnel lens, around 1.4 m away from the SPV module, enabling thermal decoupling between the CST absorber and the SPV module. This configuration allows the thermal absorber to achieve high temperatures while eliminating the need for intensive active cooling of the SPV module. In addition, practical applications of Fresnel‐lens solar concentrators require a biaxial tracker to accurately follow the sun [27].

In previous studies of semi‐transparent‐photovoltaic‐based spectral‐splitting PV‐thermal collectors, most designs placed the semi‐transparent photovoltaic device under concentrated sunlight, typically near the focal region of the solar concentrator [19, 22]. Both modeling and experimental studies have shown that, under such configurations, the photovoltaic device is still subjected to a substantial thermal load and therefore requires intensive cooling, even though part of the infrared spectrum is transmitted. An alternative approach is to position the semi‐transparent photovoltaic module above the Fresnel lens, before solar concentration, so that only the transmitted portion of the spectrum is concentrated for heat generation. This configuration avoids overheating of the photovoltaic module and enables thermal decoupling between the photovoltaic and thermal components. For example, Gupta et al. demonstrated a hybrid PV‐thermal system using a transparent cadmium telluride photovoltaic panel positioned above a Fresnel lens concentrator [28]. Liang et al. adopted a related architecture based mainly on semi‐transparent silicon photovoltaics [20]. Therefore, the present work does not claim novelty from the generic optical layout or from the general concept of using a semi‐transparent photovoltaic element as a spectral‐splitting component alone. Instead, this study focuses on the implementation and analysis of a semi‐transparent perovskite‐based SPV‐CST collector. Semi‐transparent perovskite photovoltaics offer several characteristics that are particularly attractive for this configuration [29]. Compared with silicon photovoltaics, their larger and compositionally tuneable bandgap allows the absorption edge to be shifted toward shorter wavelengths, enabling high‐energy photons to be harvested for electricity generation while transmitting a larger fraction of the near‐infrared spectrum for thermal harvesting. Moreover, recent semi‐transparent perovskite cells have demonstrated a favorable combination of high electrical efficiency and near‐infrared transmittance. In the present module, the 1.6 eV bandgap corresponds to an absorption‐edge wavelength of approximately 775 nm, allowing the transmitted near‐infrared spectrum to be subsequently concentrated for heat generation. These properties make semi‐transparent perovskites particularly promising for spectrally splitting electricity and heat generation within the SPV‐CST configuration.

### 2.2 Characteristics of the Key Components

Perovskites have emerged as a promising material for solar energy harvesting, with a tuneable bandgap [30, 31, 32]. We fabricated and tested a small‐sized perovskite module (4 cm^2^) comprised of 7 monolithically‐interconnected solar cells [33]. The structure of the SPV fabricated in our lab involves the layer structure glass/indium tin oxide (ITO) / [2‐(9H‐carbazol‐9‐yl)ethyl]phosphonic acid (2PACz) / Cs_0.17_FA_0.83_Pb(I_0.92_Br_0.08_)_3_ / lithium fluoride (LiF) / fullerene (C_60_) / bathocuproine (BCP) / tin dioxide (SnO_2_) / indium zinc oxide (IZO). The ITO serves as a transparent front electrode. The 2PACz layer acts as a self‐assembled hole transport layer to enhance hole extraction and interfacial contact. The Cs_0.17_FA_0.83_Pb(I_0.92_Br_0.08_)_3_ perovskite functions as the photoactive absorber, combining high efficiency with improved thermal and chemical stability. The LiF interlayer passivates interface defects, followed by C_60_ as an electron transport layer, while the IZO top electrode provides high infrared transmittance and serves as the transparent rear contact. The module achieved a power conversion efficiency of 14.2% with a temperature coefficient of ‐0.23%/K, as detailed in Figure S1. For this study, it is assumed that the large‐scale module exhibits similar optical and electrical performance. The optical losses in large‐scale modules caused by busbars and blank areas (the frame around the edge of the module) are also ignored. An exploded view of the SPV‐CST collector is presented in Figure 2a. Incident sunlight sequentially interacts with the SPV module, Fresnel lens, and CST absorber, enabling efficient spectral splitting and energy conversion.

**FIGURE 2:**
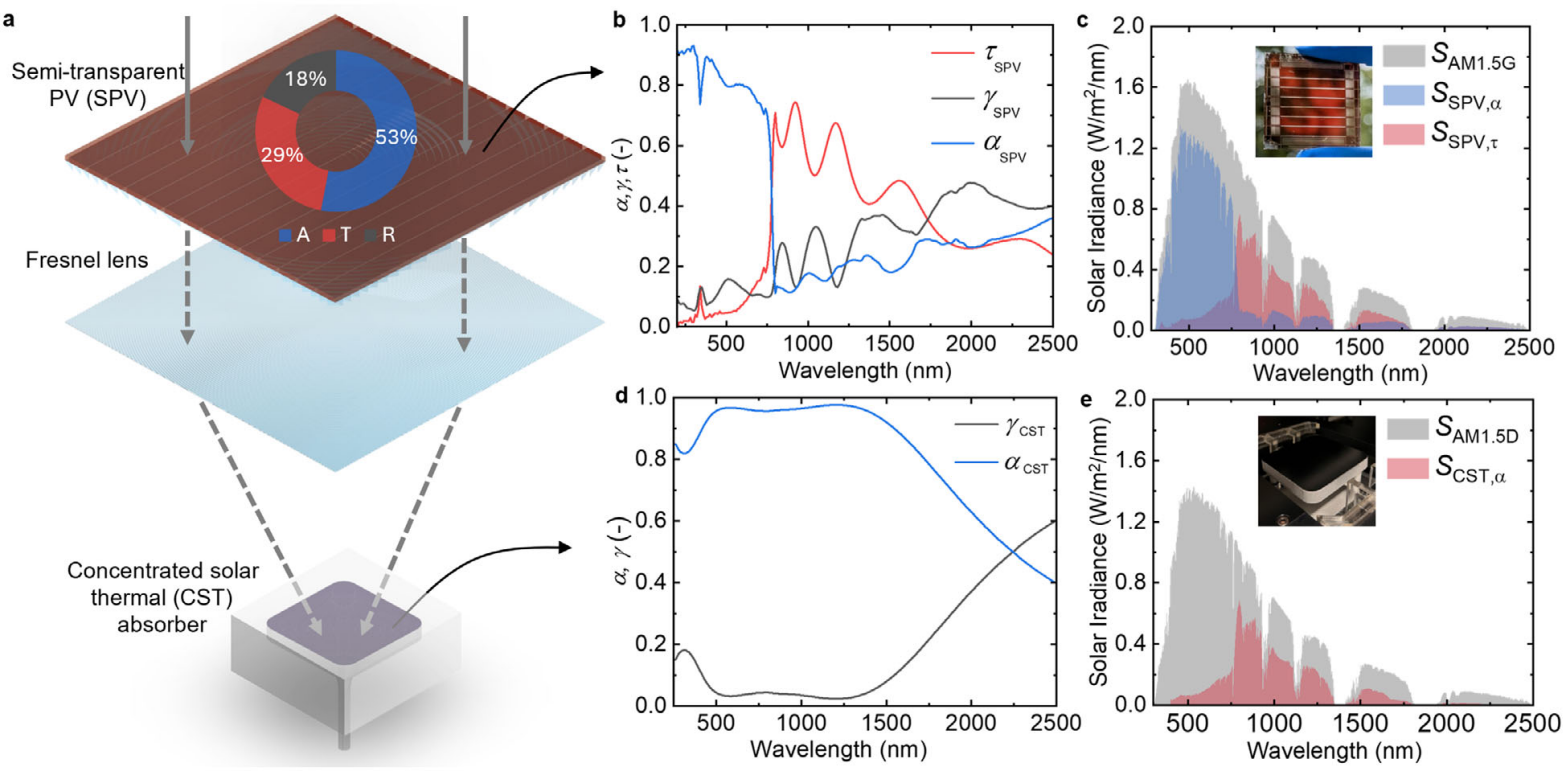
Key properties of the components in the SPV‐CST collector. (a) Component breakdown of the SPV‐CST collector, showing the spectral‐weighted average absorptance, transmittance, and reflectance of the SPV module. (b) Wavelength‐dependent absorptance, reflectance, and transmittance of the SPV module. (c) Spectral distribution of sunlight absorbed and transmitted by the SPV calculated using AM1.5G accounting for both direct and diffuse sunlight. (d) Wavelength‐dependent absorptance and reflectance of the CST absorber. (e) The spectral distribution of the solar spectrum (direct component only) absorbed by the CST absorber.

Perovskite exhibits high absorptance for wavelengths shorter than 775 nm, corresponding to its bandgap (∼1.6 eV), while also exhibiting parasitic absorption in the infrared region (due to free‐carrier absorption in the TCO layers), as shown in Figure 2b. The SPV module transmits a significant portion of the infrared spectrum but reflects a fraction of it due to reflection losses at the transparent conductive oxide interfaces. The overall spectrally‐weighted absorptance, transmittance, and reflectance of the SPV module are 53%, 29%, and 18%, respectively, calculated using the AM1.5G solar spectrum including both direct and diffuse sunlight (with the direct sunlight accounting for ∼90% and the diffuse for ∼10%), as summarized in Figure 2a. Parasitic infrared absorption in TCO electrodes can be reduced by lowering the free‐carrier concentration while maintaining high carrier mobility. Hydrogenated indium oxide (IO: H) is a promising approach because hydrogen incorporation can improve this balance and significantly reduce near‐infrared free‐carrier absorption compared with conventional indium tin oxide (ITO), while retaining sufficient electrical conductivity. This strategy could increase the near‐infrared transmittance available for subsequent thermal harvesting [34]. Spectral distribution of sunlight absorbed and transmitted by the SPV module is shown in Figure 2c. The SPV module effectively splits the solar spectrum at 775 nm. A Fresnel lens, with a geometric concentration ratio of ∼99, is positioned below the SPV module to focus the transmitted light. The transmittance of the Fresnel lens as a function of wavelength is detailed in Figure S2, with a spectrally weighted transmittance of 88.5% for the spectrum passing through the SPV module. In this study, the incident light is assumed to be normal to the Fresnel lens.

The CST absorber measures 10 × 10 × 1.2 cm and is equipped with two inlet and outlet tubes, each with a length of 7 cm. The inlet tube branches into a central straight channel and symmetrically bending channels that diverge from the center. These channels converge at the outlet tube. This built‐in channel configuration efficiently enhances heat transfer between the solid domain and the fluid flowing within the channels. Aluminum is selected as the material for the CST absorber due to its high thermal conductivity. The top surface is coated with a commercially available low‐emissivity solar thermal coating, offering high absorptivity over a wide range of wavelengths, as shown in Figure 2d. The absorptance exceeds 98% for wavelengths between 500 and 1500 nm and gradually decreases to 40% at 2500 nm. However, the solar irradiance transmitted by the SPV module is relatively low for wavelengths above 1500 nm and negligibly small beyond 1800 nm, indicating minimal optical loss for the CST absorber. The spectrum absorbed by the CST absorber, shown in Figure 2e, reaches 95% (only the direct spectrum absorbed by the CST absorber). The emissivity of the CST absorber across long wavelengths (2–20 µm), shown in Figure S3, remains below 0.2 over a wide range, effectively reducing thermal radiation losses. This low emissivity is a critical characteristic for enhancing the thermal efficiency of the CST absorber.

The relatively high reflectance of the SPV module in the near‐infrared region originates mainly from refractive‐index mismatch and optical interference at interfaces within the semi‐transparent device, particularly those associated with the transparent conductive oxide layers. These losses could be reduced through optical‐management strategies such as anti‐reflection coatings, optimization of transparent electrodes and interlayers, graded‐index coatings, and refractive‐index matching between the SPV module and Fresnel lens. Interference‐related reflection may also be reduced by introducing controlled roughness or a slightly wedge‐shaped substrate. However, because the SPV‐CST design requires precise light concentration, the impact of such modifications on optical focusing should be carefully evaluated. MgF_2_ anti‐reflection coatings applied to the outer glass surface of PV modules and transparent‐electrode optical engineering have previously been demonstrated to reduce reflection losses in perovskite and semi‐transparent perovskite solar cells, while broadband graded‐index coatings provide further potential for suppressing reflection over the visible‐to‐infrared spectrum [35, 36, 37]. Based on the measured spectrum of the present SPV module, the AM1.5G‐weighted reflectance over 780–2500 nm is 26.7%, corresponding to approximately 11.4% of the total incident solar energy. Assuming that the reduction in reflected power is converted into additional transmission while the absorptance remains unchanged, every 10% relative reduction in infrared reflectance would increase the transmitted solar‐energy fraction by approximately 1.1% (absolute value). Accordingly, the transmitted fraction would progressively increase from the present 28.6% toward an ideal upper limit of approximately 40.0% if infrared reflection were completely eliminated. Therefore, reducing infrared reflection represents an important route for further improving the thermal output of the SPV‐CST collector.

### 2.3 Modeling and Theoretical/Experimental Validation

To evaluate and optimize the performance of the SPV‐CST collector, this study develops a comprehensive model to simulate its optical‐thermal performance. The comprehensive simulation integrates two primary physics: ray optics and conjugate heat transfer and fluid dynamics, to model solar energy interaction, thermal management, and fluid flow dynamics. The analysis provides an integrated assessment of optical and thermal efficiencies, with electrical efficiency indirectly derived from temperature results. The ray optics simulation is applied to the SPV module, Fresnel lens, glazing, and solar thermal coating. Given that the device's geometric structure is significantly larger than the wavelengths of incident solar radiation, propagating waves are treated as rays that can undergo reflection, refraction, or absorption. The measured optical properties of the perovskite module are directly used. Heat transfer mechanisms, including conduction, convection, and radiation, are applied across all device domains and are detailed in Figure S4. To minimize heat losses to the ambient, the thermal compartment is insulated with glass wool, reducing both convective and radiative losses. Detailed modeling methodologies are provided in the Methods section. Key parameters used in the SPV‐CST modeling are summarized in Table 1. Optical properties and thermal emissivity are derived from the above experimental results on tested samples, while thermal insulation characteristics and convection parameters are obtained from the literature.

**TABLE 1:** Key parameters for the hybrid SPV‐CST solar collector.

Parameter	Value	Data source
*α*_SPV_ Absorptance of SPV	53.0%	Measured
*τ*_Lens_ Transmittivity of Fresnel lens	88.5%	Measured
*τ*_glass_ Transmittivity of glazing glass above CST	92.0%	Measured
*Α*_CST_ Absorptivity of CST	94.8%	Measured
*ε*_SPV_ Emissivity of SPV	0.90	Measured
*ε*_CST_ Emissivity of CST	0.23	Measured
*ε*_glass_ Emissivity of glazing glass above CST	0.90	Measured
*ε*_PMMA_ Emissivity of Fresnel lens	0.90	Measured
*ε*_wall_ Emissivity of the inner wall coating	0.04	Measured
*δ*_ins_ Thickness of thermal insulation	0.03 m	Design setting
*k*_ins_ Insulation layer’ thermal conductivity	0.03 W/m.K	Ref. 40
*υ*_wind_ Wind speed	1 m/s	Design setting
*h*_cv,wind_ Convective heat transfer coefficient due to wind	7.40 W/m^2^.K	Ref. 42
*U*_ins_ Convective heat transfer coefficient through insulation	0.88 W/m^2^.K	Ref. 42
*β* Temperature coefficient of SPV	−0.23%/K	Measured
*L*_F_ Focus distance of the Fresnel lens concentrator	1.4 m	Design setting
*A*_S_ Solar receiving area	1 m^2^	Design setting
*A_CST_* Concentrated solar‐thermal receiving area	100 cm^2^	Design setting

The performance of the SPV‐CST collectors is characterized by the electrical and thermal efficiency. The electrical efficiency η_el_ is obtained from the following equation [38]:

(1) $$\eta_{\mathrm{el}}=\eta_{\mathrm{el},0}\;\left(1+\beta \left(T_{\mathrm{cell}}-T_{\mathrm{ref}}\right)\right)$$

where η_el,0_ represents the electrical efficiency under standard test conditions (*T*_ref_ = 25°C, *I*_AM1.5G_ = 1000 W/m^2^), β is the temperature coefficient of the PV cell. The module achieved a power conversion efficiency of 14.2% with a temperature coefficient of ‐0.23%/K, as detailed in Figure S1. The thermal efficiency is defined as the ratio of useful generated heat to total incident solar irradiance on the collector, given as following [39]:

(2) $$\eta_{\mathrm{th}}=\frac{\dot{m}\times \;C_{p}\times \left(T_{\mathrm{out}}-T_{\mathrm{in}}\right)}{I_{\mathrm{AM}1.5G\;}\times A_{S}}$$

where *ṁ* is the mass flow rate, *C*_p_ is the specific heat capacity of the fluid, *T*_in is_ the temperature of the fluid at the inlet, *T*_out_ is the temperature at the outlet of the collector, and *A*_S_ is the solar receiving area (i.e., area of the solar perovskite module or the Fresnel lens).

The underlying physics used in the modeling, namely, ray optics and conjugate heat transfer and fluid dynamics, are validated both theoretically and experimentally. To ensure the quality of the mesh and the adequacy of the rays in the optical model, an additional optical simulation is performed under ideal conditions, where the transmittances of the lens and glass are set to 100%, and the absorptance of the solar thermal absorber is assumed to be 100%. The measured transmittance, absorption curves for SPV (Figure 2b) are used. The simulated solar power at the SPV and CST (*P*_SPV_ and *P*_CST_) is then compared with the theoretical values, which are calculated using the below equations:

(3) $$P_{\mathrm{SPV}\_\mathrm{theo}}=\int_{300\;\mathrm{nm}}^{2500\;\mathrm{nm}}\alpha_{\mathrm{SPV}}\left(\lambda \right)I_{\mathrm{AM}1.5G}\left(\lambda \right)d\lambda \;$$

(4) $$P_{\mathrm{CST}\_\mathrm{theo}}=\int_{300\;\mathrm{nm}}^{2500\;\mathrm{nm}}\tau_{\mathrm{SPV}}\left(\lambda \right)\tau_{\mathrm{FL}}\left(\lambda \right)\tau_{\mathrm{glas}}\left(\lambda \right)\alpha_{\mathrm{CST}}\left(\lambda \right)I_{\mathrm{AM}1.5D}\left(\lambda \right)d\lambda \;$$

The simulated power absorption values for SPV and CST are 520 and 264 W, respectively, in agreement with the theoretical values of 520 and 264 W.

The conjugated heat transfer and fluid dynamics model in the CST absorber is a critical and complex component of the SPV‐CST collector. The internal CST absorber is modeled using conjugated heat transfer and fluid dynamics because the built‐in channel geometry, nonuniform concentrated heat flux, and coupled solid‐fluid heat transfer cannot be captured adequately by a single effective heat‐transfer coefficient. By contrast, literature‐based heat‐transfer coefficients are used only for external boundary conditions, where such treatment is appropriate. To validate this model, a solar thermal absorber was fabricated and tested, as shown in Figure 3a,b. The solar thermal absorber was manufactured using additive aluminium alloy and designed with the same geometry as the CST absorber in the SPV‐CST collector. The solar thermal absorber was mounted within a polymethyl methacrylate (PMMA) chamber and tested under a solar simulator. The purpose of this experiment was to validate the conjugated heat transfer and fluid dynamics model. For practicality and to meet validation requirements, certain adjustments were made during the testing. Liquid water, with a flow rate ranging from 1.5 to 3.8 kg/h (1.5, 2.2, 3.1, and 3.8 kg/h), was used as the heat transfer fluid instead of heat transfer oil. The top surface of the absorber was coated with matte black paint, as shown in Figure 3b. The chamber was filled with air instead of being vacuum‐sealed, and insulation layers were not applied to the outer surface. The detailed optical properties of the matte black coating are provided in Figure S5. In the modeling, the same parameters as those of the real solar thermal absorber were applied, as detailed in Table S1. A comprehensive description of the experimental setup and modeling validation can be found in the Methods section.

**FIGURE 3:**
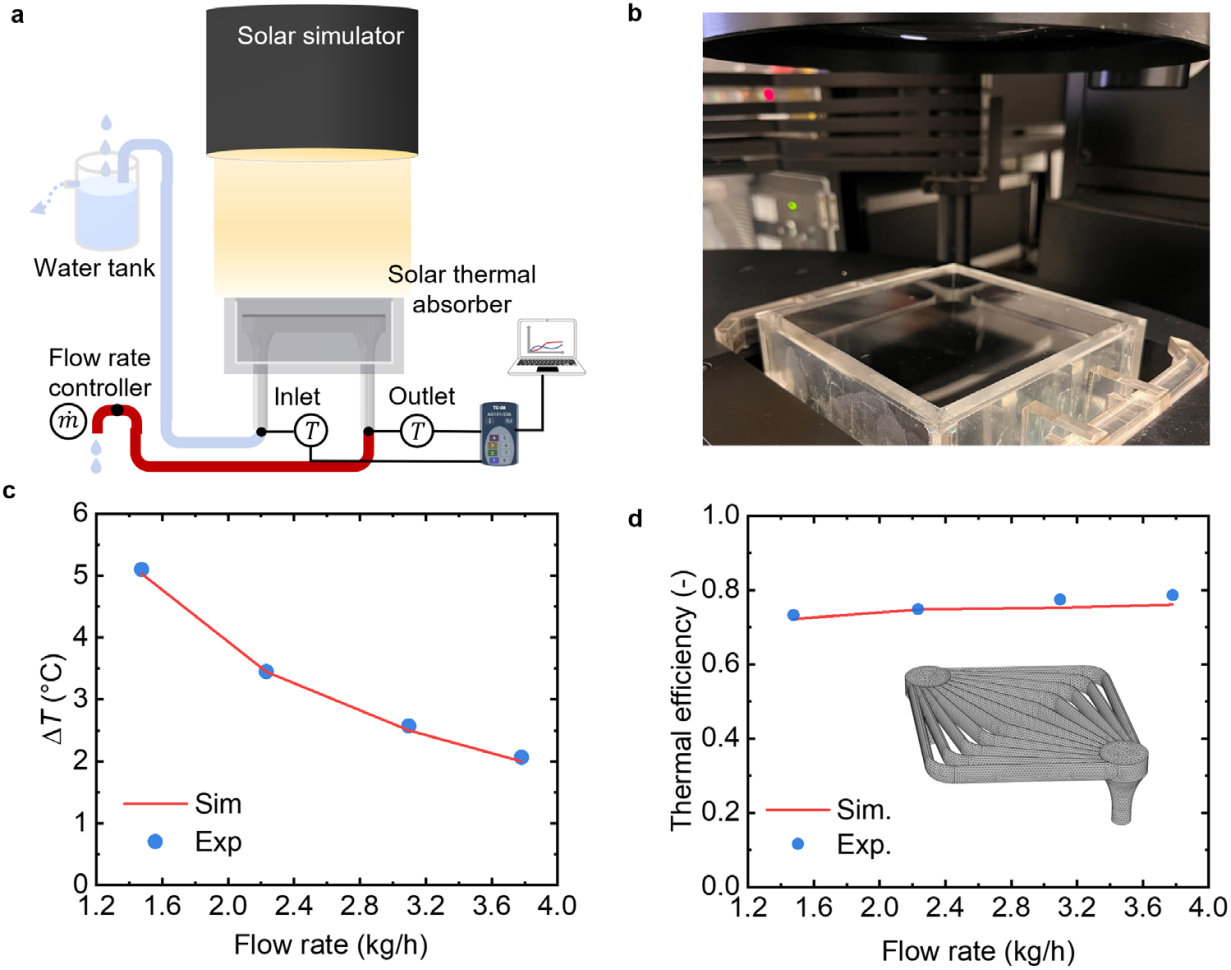
Experimental process for validating the developed model. (a) Schematic diagram of the experimental setup used for testing. (b) Photograph of the solar thermal collector under the solar simulator. (c) Comparison of experimental and simulated temperature differences between the inlet and outlet of the solar thermal absorber across various flow rates. (d) Variation of thermal efficiency with flow rate.

The experimental data for the temperature difference between the inlet and outlet of the solar thermal absorber, as a function of flow rate, is plotted in Figure 3c alongside the simulated data. The inlet temperature is 16.7, 16.5, 16.6, and 16.6°C at mass flow rates of 1.5, 2.2, 3.1, and 3.8 kg/h, respectively, and detailed steady‐state experimental data are shown in Figure S6. The thermal efficiency of the solar thermal absorber is shown in Figure 3d. A comparison of these experimental results with the simulation data demonstrates that the developed model accurately replicates the thermal performance of the solar thermal collector, with a maximum variation of less than 4%. Figure 3d compares the experimental and simulated thermal efficiencies over the accessible flow‐rate range used for model validation. It is not intended to determine the upper limit of thermal efficiency. These findings validate the reliability of the model, as it employs the same heat transfer and fluid dynamics physics used in the concentrating device. Overall, the agreement between the analytical calculation, simulation, and experiment supports the consistency of the modeling framework for the present SPV‐CST configuration and operating conditions.

### 2.4 Performance Assessment of the SPV‐CST Collector

In this section, the optical, thermal, and electrical performance of the SPV‐CST collector is investigated and analyzed. Geometrical optics simulations are used to assess the ray tracing within the SPV‐CST collector and the solar flux distribution on the surface of the CST absorber, as shown in Figure 4a. In this study, AM1.5G is used as the input solar irradiance with a total of 1000 W/m^2^, comprising ∼900 W/m^2^ direct irradiance that can be concentrated and ∼100 W/m^2^ diffuse irradiance that cannot be concentrated. As discussed earlier, the SPV utilizes both direct and diffuse components, whereas the CST can only harness the concentrated direct component. The ray trajectories confirm the efficient concentration of solar rays by the Fresnel lens onto the CST absorber, with no stray rays observed. The optimized positioning of the solar thermal absorber, 1.4 m from the lens, ensures effective capture and uniform distribution of rays across the absorber surface. The lens produces a nonuniform square intensity distribution on the thermal absorber, with the highest intensity at the center gradually decreasing toward the edges. This illumination effectively covers nearly the entire area of the thermal absorber. The integrated solar intensity over the CST absorber is 2.1 × 10^4 ^W/m^2^. This efficiency accounts for absorption by the SPV module and optical losses through the Fresnel lens, glazing glass on the CST, and reflections from the CST absorber. The solar energy flux absorbed by the SPV module is measured at 539 W/m^2^. A portion of this absorbed energy is converted to electricity based on the electrical efficiency of the SPV module, while the remainder is dissipated as waste heat within the SPV module.

**FIGURE 4:**
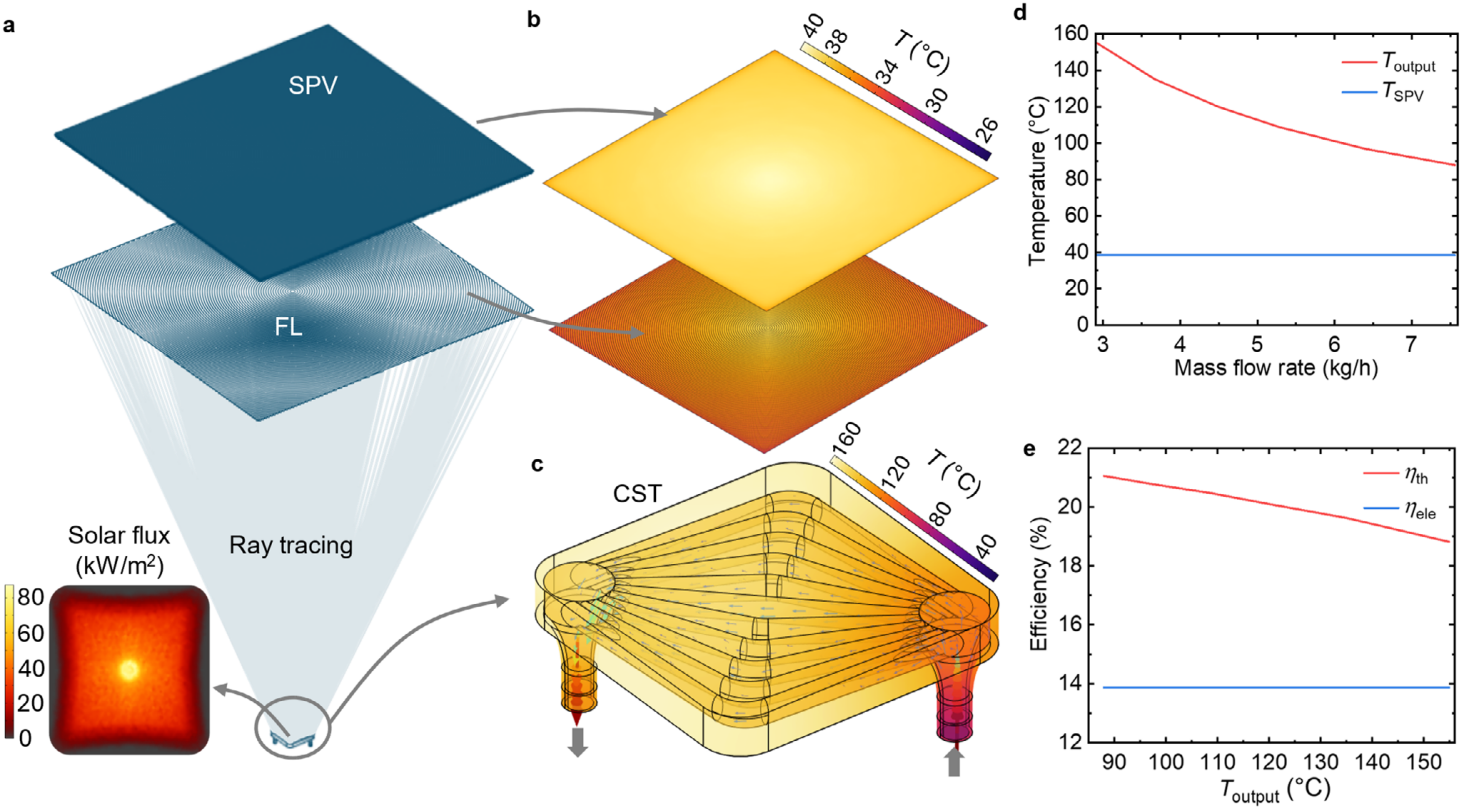
Performance of the SPV‐CST collector. (a) Ray trajectories demonstrating the efficient concentration of solar rays onto the CST absorber, with a square intensity distribution showing higher concentration at the centre produced by the Fresnel lens. (b) Temperature distribution of the SPV module and Fresnel lens. (c) Temperature distribution and fluid streamlines within the CST absorber under the following conditions: total incident intensity of 1000 W/m2, ambient temperature of 25°C, wind speed of 1 m/s, inlet temperature of 30°C, and flow rate of 4 kg/h. (d) Output temperatures of the CST absorber as a function of flow rate. (e) Thermal and electrical efficiencies of the SPV‐CST collector as a function of output heat temperatures.

The temperature distributions in the SPV module and Fresnel lens are shown in Figure 4b, while the temperature distribution in the CST absorber under conditions of a total direct incident intensity of 1000 W/m^2^, ambient temperature of 25°C, wind speed of 1 m/s, inlet temperature of 30°C, and flow rate of 4 kg/h is presented in Figure 4c. The fluid is gradually heated from 30°C to 135.1°C by the CST, while the top surface of the CST (solar absorbing surface) has a more uniform temperature distribution within around 120°C to 160°C. The results clearly demonstrate that the SPV module is thermally decoupled from the CST absorber. The SPV is under non‐concentrated sunlight, while only the CST is under concentrated sunlight. This thermal decoupling allows the CST absorber to operate at elevated temperatures without negatively impacting the SPV module or requiring active cooling systems. The average temperature of the CST absorber is 146°C, with the outlet temperature of the heat transfer oil recorded at 135°C. In contrast, the SPV module maintains a low operating temperature of 38.5°C.

The CST absorber exhibits a temperature gradient along the fluid flow path, as shown in Figure 4c. The temperature within the fluid domain is lower than that of the solid domain, due to thermal resistance during heat transfer from the solid absorber to the heat transfer fluid. The arrows within the fluid domain illustrate the fluid flow patterns and velocity variations as the fluid moves through the tubes and channels of the thermal absorber.

The strength of the modeling lies in its ability to analyse system dynamics that cannot be fully captured through experiments. The velocity distribution, shown in Figure S7, indicates that the fluid velocity is highest at the centre of the channel and gradually decreases toward the channel walls due to the no‐slip condition and viscosity effects, resulting in a parabolic velocity profile characteristic of laminar flow. The temperature distribution, also presented in Figure S8, illustrates the evolution of the fluid temperature as it flows through the CST absorber. The results reveal a uniform temperature distribution at the outlet, which is crucial for practical applications. A stable outlet temperature ensures consistent performance and reliability in various industrial processes, such as chemical reactions and material processing.

The average outlet temperature of the CST absorber (*T*_out_​) and the average SPV module temperature (*T*_SPV_) as a function of flow rate are plotted in Figure 4d. The outlet temperature increases from 88.0°C to 154.9°C as the mass flow rate decreases from 7.5 to 2.9 kg/h, demonstrating the SPV‐CST collector's capability to generate thermal energy over a wide range of temperatures to meet diverse thermal demands. Despite the variation in outlet temperature, the operating temperature of the SPV module remains nearly constant at approximately 38.5°C, unaffected by changes in the mass flow rate. Detailed temperature distributions within the CST absorber for flow rates ranging from 2.9  to 7.5 kg/h are shown in Figure S9. The thermal and electrical efficiencies of the SPV‐CST collector are plotted as a function of output temperature in Figure 4e for the specified flow rate range. The thermal efficiency is calculated from the energy balance in Equation (2). While the thermal efficiency varies between 18.8% and 21.0%, the electrical efficiency remains consistent at 13.8%.

The SPV‐CST concept should not be interpreted as a universal replacement for standalone photovoltaic modules, solar‐thermal collectors, or separated photovoltaic‐plus‐thermal systems. If the objective is only to maximize electricity generation, opaque high‐efficiency photovoltaic modules remain preferable. Similarly, when sufficient installation area is available, separate photovoltaic and thermal collectors may offer a simpler and more mature solution. The value of the SPV‐CST configuration lies instead in co‐harvesting electricity and heat from the same solar aperture (with limited area) while maintaining thermal decoupling between the photovoltaic and thermal components. The performance benefit therefore depends on the efficiency penalty associated with semi‐transparency, the useful thermal output recovered from the transmitted spectrum, the direct irradiance level, the diffuse fraction, and the required heat‐delivery temperature. For example, if an opaque photovoltaic module provides a 3% absolute electrical‐efficiency advantage over a semi‐transparent module, using this additional electricity to drive a heat pump with a COP of 3 would yield an equivalent thermal output of around 9% of the incident solar energy. This provides an illustrative benchmark for evaluating the thermal contribution of the SPV‐CST collector. Under favorable direct‐irradiance conditions, the simulated thermal output of the SPV‐CST collector can exceed this value, although the advantage decreases under highly diffuse conditions or when the photovoltaic efficiency penalty becomes larger. Therefore, the present study positions the SPV‐CST collector as an early‐stage co‐harvesting concept for aperture‐limited applications with simultaneous electricity and heat demand, rather than as a universally superior alternative to mature separated systems.

In practical applications, the performance is influenced by weather conditions, particularly solar irradiance and ambient temperature. As shown in Figure S10a, the output temperature decreases from 135.1°C to 74.5°C as solar irradiance drops from 1000 to 400 W/m^2^ at a constant mass flow rate of 4 kg/h. Despite this significant reduction in output temperature, the thermal efficiency remains stable at approximately 19.6%. Figure S10b also demonstrates that the thermal efficiency decreases only slightly from 19.6% to 19.2% as the ambient temperature drops from 30°C to 0°C. This minimal change in thermal efficiency can be attributed to the effective thermal insulation techniques employed in the collector, including the vacuum gap, low‐emissivity coating, and thermal insulation layers. The thermal energy can be stored for practical applications [43].

These results demonstrate that the adopted spectral splitting approach, utilizing an SPV module, effectively achieves thermal decoupling between the CST and SPV components. This decoupling is evident from the temperature distribution and analyzed data, which clearly indicate that the SPV module's temperature remains independent of the high‐temperature solar thermal absorber. This finding highlights the effectiveness of the developed design in overcoming the trade‐off between thermal and electrical efficiencies. The electrical efficiency remains entirely unaffected by variations in the thermal collector, enabling the maximization of solar irradiance utilization and, consequently, enhancing the overall system efficiency.

### 2.5 Analysis of the Potential of the SPV‐CST Collector

It should be noted that the electrical efficiency adopted in this study is based on the perovskite PV module fabricated in our laboratory. The main purpose of this work is to evaluate the feasibility and performance of the SPV‐CST concept. The electrical performance of perovskite solar modules still has substantial room for improvement, and ongoing progress in perovskite photovoltaics provides an opportunity to further enhance the performance of the SPV‐CST collector.

Recent semi‐transparent perovskite PV cells have already achieved efficiencies of 22.0% [44]. To analyze the potential of the SPV‐CST collector with improved SPV performance, we investigate and compare three different semi‐transparent perovskite PV cells in this section: the one developed in our laboratory and used in the previous sections (denoted as SPV1), and two additional semi‐transparent perovskite PV cells reported in the literature (denoted as SPV2 [44] and SPV3 [45]). Figure 5a shows the measured transmittance spectra of SPV1, SPV2, and SPV3. These three PV cells have similar bandgaps, corresponding to wavelengths of around 770 to 800 nm. All three exhibit low transmittance in the in‐band wavelength range and high transmittance in the out‐of‐band wavelength range. For SPV2 and SPV3, the transmittance data beyond 1300 nm are not available in the literature. Therefore, for wavelengths beyond 1300 nm, the transmittance spectrum of SPV1 is used as an approximation for SPV2 and SPV3 in the SPV‐CST modeling.

**FIGURE 5:**
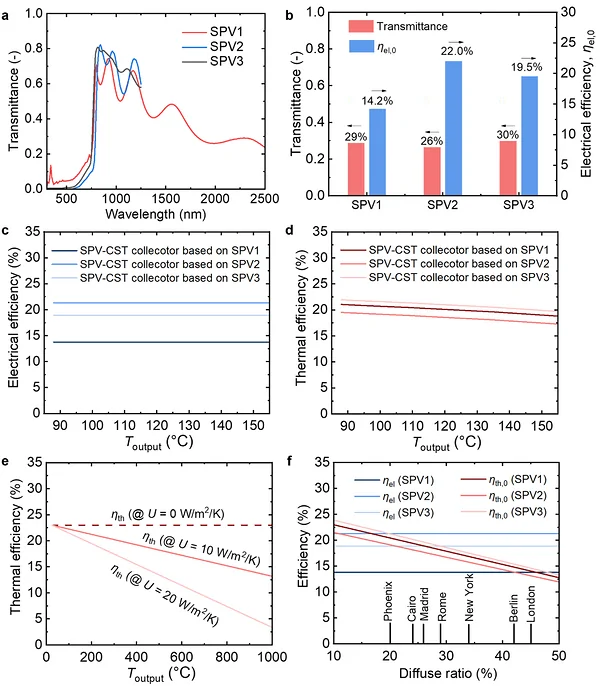
Analysis of the potential of the SPV‐CST collector. (a) Measured transmittance spectra of three semi‐transparent perovskite photovoltaic cells used for comparison in this study: SPV1 from this work, and SPV2 and SPV3 from the literature. (b), Spectral‐weighted average transmittance and electrical efficiency of SPV1, SPV2, and SPV3. (c), Simulated electrical efficiencies of the SPV‐CST collectors based on SPV1, SPV2, and SPV3 as a function of output temperature. (d) Simulated thermal efficiencies of the SPV‐CST collectors based on SPV1, SPV2, and SPV3 as a function of output temperature. (e) Thermal efficiency of the SPV‐CST collector based on SPV1 as a function of output temperature for different heat loss coefficients (*U* = 0, 10, and 20 W/m^2^/K), representing ideal, good, and normal thermal insulation, respectively. (f) Electrical and thermal efficiencies of the SPV‐CST collector with SPV1, SPV2, and SPV3, as a function of diffuse light ratio, showing the influence of diffuse solar irradiance on system performance. Representative cities with different annual‐average diffuse fractions are marked to illustrate the relevance of the simulated range to different climatic conditions.

Figure 5b shows the spectral‐weighted average transmittance and electrical efficiency of SPV1, SPV2, and SPV3. The transmittances of SPV1, SPV2, and SPV3 are 29%, 26%, and 30%, respectively, while their electrical efficiencies are 14.2%, 22.0%, and 19.5%, respectively. SPV2 has an absolute electrical‐efficiency advantage of 7.8% over SPV1, while its transmittance is only 3% lower in absolute terms. SPV3 also shows a higher electrical efficiency than SPV1, with an absolute increase of 5.3%, while maintaining a very similar transmittance, with a difference of only around 1% in absolute terms.

We then model the SPV‐CST collectors based on SPV2 and SPV3 and compare them with the collector based on SPV1. The electrical and thermal efficiencies of the SPV‐CST collectors based on SPV1, SPV2, and SPV3 are shown in Figures 5c,d. Owing to the effective thermal decoupling between the perovskite solar cells and the solar‐thermal absorber, the electrical efficiency remains independent of the heat output temperature, and the SPV‐CST collector based on SPV2 shows the highest electrical efficiency. The electrical efficiencies of the SPV‐CST collectors based on SPV1, SPV2, and SPV3 are 13.8%, 21.3%, and 18.9%, respectively. The thermal efficiencies of the SPV‐CST collectors based on SPV1, SPV2, and SPV3 are 20.7%, 19.2%, and 21.6%, respectively, at an output temperature of 100°C. These results indicate that the electrical efficiency of the SPV‐CST collector can be further improved by using higher‐performance semi‐transparent PV cells without significantly compromising the thermal efficiency. By using SPV2 instead of SPV1, the electrical efficiency of the SPV‐CST collector increases by 7.5% in absolute terms, while the thermal efficiency decreases by only 1.5% in absolute terms. This comparison highlights the strong potential of improved semi‐transparent perovskite PV cells for enhancing the overall performance of SPV‐CST collectors.

The best opaque perovskite PV cells have achieved efficiencies above 26% [46]. However, it should be noted that semi‐transparent perovskite PV cells generally have lower electrical efficiencies than opaque ones. Semi‐transparent PV cells are not expected to reach the same power conversion efficiency as opaque cells, because the required transparent conductive oxide back contacts do not provide equally low electrical resistance and also reduce the optical path length associated with back‐side reflection. While the current record efficiency for opaque perovskite solar cells exceeds 26%, semi‐transparent perovskite PV cells have reached 22% to the best of our knowledge, as represented here by SPV2.

The bandgap wavelength is fixed at 775 nm in this study. It would also be valuable to investigate the influence of bandgap on the electrical and thermal performance of the SPV‐CST collector. However, such an analysis is not carried out here because sufficiently complete optical data across the full solar spectrum range (300–2500 nm) are not yet available in the literature for semi‐transparent perovskite cells with different bandgaps. Most semi‐transparent perovskite cells reported in the literature have bandgaps around 1.5 to 1.6 eV in order to achieve high electrical efficiency, and their optical spectra are often measured only up to 1300 nm because most studies focus on tandem PV applications. This remains an important direction for future work on SPV‐CST collectors.

We also investigate the thermal potential of the SPV‐CST collector. In the previous section, the main objective was to demonstrate thermal decoupling between the perovskite solar cells and the solar‐thermal absorber. To limit the computational cost of the conjugated heat transfer and fluid dynamics simulations, the analysis in Figure 4e is restricted to heat output temperatures up to about 150°C. However, the achievable output temperature can be considerably higher, depending on the thermal insulation performance of the solar‐thermal collector. As shown in Figure 5e, the thermal efficiency of the SPV‐CST collector is evaluated for heat loss coefficients (U‐values) of 0, 10, and 20 W/m^2^/K, corresponding to ideal insulation without heat loss, good insulation achievable with vacuum insulation, and normal insulation, respectively. Based on the energy balance, much higher output temperatures can in principle be reached if an appropriate heat transfer medium is used, such as molten salts or liquid metals.

Another advantage of the SPV‐CST collector is its ability to utilize diffuse solar radiation for electricity generation. In conventional concentrating solar collectors (e.g., concentrated PV, concentrated solar‐thermal collectors), diffuse light cannot be concentrated and is therefore not utilized. In the SPV‐CST design, by contrast, the diffuse component can still contribute to electricity generation, with only the diffuse near‐infrared component remaining unused for thermal production. In the previous section, the system performance is evaluated for a standard diffuse ratio of 10%. Here, additional simulations are conducted to examine the influence of the diffuse ratio on the electrical and thermal performance over a range from 10% to 50% for the SPV‐CST collectors based on SPV1, SPV2, and SPV3, as shown in Figure 5f. The results show that the diffuse ratio has no effect on the electrical efficiency, whereas the thermal efficiency at ambient temperature (*η*_th,0_) decreases linearly as the diffuse ratio increases. It is important to note that the diffuse fraction of natural sunlight varies substantially with geographical location and climate. For example, diffuse irradiation accounts for approximately 20%, 24%, 26%, 30%, 34%, 43%, and 45% of the annual global horizontal irradiation in Phoenix, Cairo, Madrid, Rome, New York, Berlin, and London, respectively [26]. These representative values cover much of the 10%–50% diffuse‐radiation range investigated in Figure 5f, linking the simulation conditions to a broad range of real‐world solar climates. Regions with lower diffuse fractions are more favorable for the concentrating thermal branch, whereas the SPV module can continue to harvest both direct and diffuse irradiance for electricity generation. For a broader system‐level perspective, the key performance metrics of representative thin‐film‐filter‐based, nanofluid‐based, and semi‐transparent‐PV‐based spectral‐splitting PV‐thermal collectors reported in the literature are quantitatively compared with those of the present SPV‐CST collector in Table S2.

The bandgap of semi‐transparent perovskite photovoltaics is tunable and therefore provides an additional degree of freedom for balancing electricity and thermal‐energy generation. To illustrate this fundamental trade‐off, Figure S11 compares the Shockley–Queisser photovoltaic efficiency limit with the ideal upper limit of solar energy available for thermal harvesting over bandgaps from 1.5 to 2.3 eV. As the bandgap increases from 1.5 to 2.3 eV, the theoretical photovoltaic efficiency decreases from approximately 32.1% to 16.7%, while the fraction of AM1.5D solar energy at wavelengths longer than the corresponding absorption edge increases from approximately 39.4% to 72.4%. This analysis highlights the intrinsic spectral trade‐off in selecting the SPV bandgap: increasing the bandgap shifts a larger fraction of the solar spectrum toward the thermal branch, while decreasing the bandgap increases the photovoltaic conversion potential at the expense of transmitted thermal energy.

## 3 Conclusions

This study demonstrates the potential of spectral splitting to optimize solar spectrum utilization through a novel hybrid SPV‐CST collector. The design integrates a semi‐transparent perovskite (SPV) module placed in front of a Fresnel lens to harvest global solar irradiance, while the lens focuses the transmitted direct spectrum onto a concentrated solar thermal (CST) absorber for high‐temperature thermal energy generation. The CST absorber, equipped with built‐in channels, ensures enhanced heat transfer and uniform temperature distribution. The present configuration achieves thermal decoupling between the photovoltaic module and the high‐temperature thermal receiver by placing the semi‐transparent photovoltaic module before the concentrator, so that the photovoltaic branch operates under unconcentrated sunlight while only the transmitted spectrum is concentrated for thermal harvesting. In this study, the experiment establishes the feasibility of the concept and validates the thermal model, while the full SPV‐CST performance under higher‐temperature operating conditions is evaluated using the validated multiphysics model.

The performance of the SPV‐CST collector was thoroughly evaluated using coupled optical, thermal, and fluid dynamic simulations, validated through theoretical analysis and experimental testing of a prototype under no concentration conditions. Results reveal effective thermal decoupling, maintaining the SPV module temperature at a consistent 38.5°C across all flow rates, thereby eliminating the need for active cooling. The thermal absorber achieves temperatures up to 154.9°C at a flow rate of 2.9 kg/h, while the electrical efficiency remains stable at 13.8%, independent of absorber temperature. These outcomes confirm the system's ability to maintain independent thermal and electrical performance.

Future efforts will focus on advancing perovskite photovoltaic technologies to enhance the efficiency of the SPV module and further optimize the system's overall performance. Research aimed at minimizing optical losses in the SPV module, Fresnel lens, and CST absorber will be critical for maximizing solar energy capture and utilization. Additionally, the integration of high‐efficiency, record‐breaking perovskite PV cells, with efficiencies exceeding 26%, offers the potential to elevate the system's electrical efficiency to approximately 25% with minimal impact on thermal performance.

These findings underline the proposed SPV‐CST collector's potential to provide a sustainable and efficient solution for simultaneous electricity and thermal energy generation. The design is adaptable to various operational conditions and applications, supporting the global energy sector's transition toward net‐zero emissions. By addressing key challenges in solar energy utilization, this work contributes to the advancement of hybrid solar energy systems, aligning with sustainability goals and paving the way for future innovations in renewable energy technologies.

Improving the long‐term operational stability of perovskite solar cells is critically important. Previous studies have shown that perovskite materials are susceptible to degradation under thermal cycling, humidity, and prolonged UV exposure, which can lead to phase instability, ion migration, and performance loss [47, 48, 49]. In particular, UV irradiation is known to accelerate chemical decomposition, defect formation, and interfacial degradation [50, 51]. Recent advances, including compositional engineering, surface passivation, interface optimization, and improved encapsulation strategies, have demonstrated promising progress in enhancing device stability under realistic operating conditions [51, 52]. Recent studies further show that, in semi‐transparent perovskite solar cells, stability can also be limited by degradation at the transparent‐contact interfaces [44]. In particular, lithium species from the doped hole‐transport layer can diffuse into the metal‐oxide buffer layer, accelerating fill‐factor loss and long‐term degradation. Interface engineering that converts reactive lithium species into more stable lithium oxides has enabled semi‐transparent devices with an electrical efficiency of ∼22%, > 99% shelf stability after 400 h, and > 99% operational stability for 240 h. These findings are directly relevant to the present SPV‐CST system (the SPV2 used in this study), where the long‐term robustness of transparent‐contact interfaces will be important for practical application. Future research on SPV–CST systems should therefore focus on systematic stability evaluation of semi‐transparent perovskite modules under realistic solar irradiation, ambient thermal cycling, humidity, and UV exposure, as well as on the development of durable encapsulation approaches for long‐term operation.

For practical outdoor operation, the SPV‐CST collector requires solar tracking to maintain near‐normal incidence on the Fresnel lens and preserve optical concentration performance. The results presented in this study therefore represent the performance under ideal optical alignment, as achieved in the experiments under a solar simulator. Under off‐normal incidence, the optical efficiency of the concentrating path would decrease, leading primarily to a reduction in the thermal output of the CST absorber. In contrast, the SPV module is positioned before the concentrator and can still directly harvest both direct and diffuse solar irradiance, so the photovoltaic branch is expected to be less sensitive to tracking errors than the thermal concentrating branch. Like those faced by conventional solar materials and devices, UV irradiation, humidity, thermal cycling, and soiling can affect the practical deployment and long‐term performance of the proposed SPV‐CST collector. UV‐induced degradation can be mitigated by using UV‐resistant optical materials, such as glass‐based Fresnel lenses, together with UV‐stabilized encapsulation materials such as ethylene‐vinyl acetate (EVA). The influence of humidity can be reduced through effective encapsulation and edge sealing, while degradation caused by repeated thermal cycling can be minimized through appropriate material selection and mechanically robust interfaces that accommodate thermal expansion. Particularly, soiling is another important factor for outdoor operation of the SPV‐CST collector. Dust deposition on the SPV surface reduces both photovoltaic output and the near‐infrared flux transmitted toward the thermal branch, while additional soiling on the bottom surface of the Fresnel lens further decreases the optical power reaching the CST absorber. As a first‐order sensitivity analysis, assuming spectrally uniform attenuation, equal soiling losses of 5%, 10%, and 20% on the SPV and Fresnel lens would reduce the electrical output by 5%, 10%, and 20%, respectively, and the thermal input by approximately 9.8%, 19.0%, and 36.0%. Actual soiling losses are site‐ and wavelength‐dependent, and future outdoor studies should investigate dust accumulation, spectral attenuation, cleaning frequency, and suitable anti‐soiling strategies. For the solar tracking system, tracking errors primarily affect the CST branch rather than the SPV branch. For the SPV module, the incident direct irradiance approximately follows a cosθ dependence; therefore, tracking offsets of θ = 0.5°, 1°, and 2° reduce the direct irradiance by only approximately 0.004%, 0.015%, and 0.061%, respectively. In contrast, angular deviations shift the focal region of the Fresnel lens relative to the CST absorber. For the present focal distance of 1.4 m, tracking errors of θ = 0.5°, 1°, and 2° correspond to focal shifts of approximately 1.22, 2.44, and 4.89 cm, respectively. Assuming a 10 cm receiver matching the nominal focal footprint, a first‐order two‐dimensional geometrical analysis gives corresponding spillover losses of approximately 12.2%, 24.4%, and 48.9%. In practical systems, a receiver larger than the nominal focal footprint can provide additional optical acceptance tolerance; for example, a 15 cm receiver can accommodate approximately 1° tracking deviation with negligible spillover in this simplified analysis. Receiver sizing therefore represents a trade‐off between tracking tolerance and thermal heat losses.

From a practical perspective, the SPV‐CST collector studied here can be regarded as an upgraded Fresnel‐lens‐concentrated solar‐thermal collector, in which a semi‐transparent perovskite module is added above the Fresnel lens to enable simultaneous electricity generation. In this sense, the additional system cost is mainly associated with the semi‐transparent perovskite module and its integration, while the concentrating solar‐thermal part remains largely based on an existing collector architecture. A first‐order estimate therefore suggests that the capital cost of the SPV‐CST system may be approximated as the sum of a Fresnel‐lens‐concentrated solar‐thermal collector and a semi‐transparent perovskite module. Based on this view, the overall payback time of the SPV‐CST collector is expected to be on the order of that of the two subsystems, although a more rigorous estimate will require detailed analysis of integration cost, system lifetime, maintenance, and the application‐specific values of electricity and high‐temperature heat. On the other hand, the SPV‐CST collector offers the ability to simultaneously generate electricity and high‐temperature heat from the same collector area, which may provide practical and economic advantages in applications where both outputs are required and available installation area is limited. This will be an important subject for future techno‐economic analysis. SPV‐CST collector occupies a different application space from both separate PV plus CST systems and conventional non‐concentrating PV/T collectors. Separate PV and CST systems offer greater technological maturity and allow independent optimization of electricity and thermal generation, but generally require separate collector areas and balance‐of‐system components. In contrast, the SPV‐CST architecture co‐generates electricity and medium‐ to high‐temperature heat from the same solar aperture, which can be advantageous where installation area is limited and both energy outputs are required. Conventional non‐concentrating hybrid PV‐thermal collectors offer simpler system architectures and are well suited to low‐temperature heat applications, but their thermal output temperature is constrained by the thermal coupling between the PV module and thermal absorber. The hybrid SPV‐CST concept in this study is therefore attractive under high‐direct‐irradiance conditions with simultaneous demand for electricity and medium‐ to high‐temperature heat, whereas it is likely to be less favorable when installation area is abundant, irradiance is highly diffuse, only one energy product is required, or the additional costs of semi‐transparent perovskite modules and optical integration outweigh the benefits of co‐generation.

The present study combines experimental characterization and model‐supported system analysis. The semi‐transparent perovskite photovoltaic module is characterized experimentally through its measured optical transmittance, absorptance, electrical efficiency, temperature coefficient, and operating temperature under solar‐simulator illumination. The CST absorber is experimentally tested under controlled conditions because it represents the most complex part of the coupled model, involving optical absorption, heat conduction, convective heat transfer to the working fluid, and fluid dynamics in the internal flow channel. The full SPV‐CST collector performance is then assessed using the measured SPV properties, measured optical properties of the collector components, and the validated CST absorber model. An integrated experimental demonstration of the complete SPV‐CST collector would provide further validation of the full architecture and is an important direction for future work.

Although the present study evaluates the SPV‐CST concept at a collector aperture of approximately 1 m^2^, the experimentally fabricated semi‐transparent perovskite module in this study from our lab is currently limited to 4 cm^2^. However, commercial‐scale perovskite photovoltaic modules with dimensions up to approximately 1.2 × 2.4 m have already been demonstrated [53], indicating substantial progress in large‐area perovskite module fabrication, although further development is still required for high‐performance semi‐transparent modules. A key step toward practical deployment is therefore the scalable fabrication of large‐area, stable semi‐transparent perovskite modules and their optical integration with Fresnel‐lens‐based thermal collectors. Further upscaling can follow a modular approach, in which multiple SPV‐Fresnel‐CST units are arranged in arrays with shared tracking structures, thermal manifolds, and electrical infrastructure. At larger scales, alternative concentrating architectures, such as linear Fresnel reflector systems integrated with semi‐transparent perovskite photovoltaics, may also provide a promising pathway for simultaneous electricity and thermal‐energy generation [21]. Future development should therefore focus on large‐area SPV fabrication, long‐term outdoor stability, optical alignment and integration, thermal‐system integration, tracking strategies, and system‐level techno‐economic assessment.

## 4 Methods

### 4.1 Experimental Method

The measurements are conducted using a dual‐lamp continuous solar simulator (Wacom, class AAA) that replicates sunlight intensity and spectral distribution under standard test conditions (AM1.5). The incident light intensity is calibrated with a pyranometer. A gravity‐driven water flow system, controlled by the elevation of the water tank, eliminates the need for a pump. Flow rate is regulated at the outlet by a valve and measured manually with a measuring cylinder and stopwatch. K‐type thermocouples monitor inlet and outlet water temperatures, recorded along with ambient temperature using a data logger (PicoLog). Experimental results are validated against simulation data using inlet and ambient temperatures as input parameters. The measurements of the reflection and transmission properties were carried out using a UV–vis–NIR spectrophotometer (Agilent Cary 7000).

### 4.2 Modeling Methods

This study employs COMSOL Multiphysics 6.0 to simulate the optical‐thermal performance of a semi‐transparent photovoltaic (SPV) and concentrated solar thermal (CST) collector. Using the finite element method (FEM), the simulation involves two main physics: ray optics, conjugated heat transfer and fluid dynamics, to model solar energy interaction, thermal management, and fluid flow dynamics. In addition to the main physics interfaces, the simulation includes three coupling multiphysics interfaces: ray heat source, non‐isothermal flow, and heat transfer with surface‐to‐surface radiation. This ensures an integrated assessment of optical and thermal efficiencies, with the electrical efficiency indirectly derived from temperature results. A two‐step simulation process is used: the first step involves a time‐dependent optical simulation to assess the concentration performance of the Fresnel lens and determine solar power received by SPV and CST. The results obtained from the first step are fed into the second step, which involves steady‐state heat transfer and fluid dynamics analysis to study the thermal response and optimize system performance.

The model inputs and boundary conditions are defined from measured data and tabulated material parameters. The wavelength‐dependent transmittance and absorptance of the semi‐transparent perovskite photovoltaic module are taken from the measured spectra shown in Figure 2, while the optical properties of the Fresnel lens, glazing glass, absorber coating, and insulation materials are summarized in Table 1. The electrical efficiency of the semi‐transparent perovskite module and its temperature coefficient are obtained from the measured photovoltaic performance shown in Figure S1. Thermal boundary conditions, including ambient temperature, wind speed, external convective heat transfer, inlet water temperature, and mass flow rate, are specified according to the experimental or simulation conditions described in the main text and Table 1. The thermophysical properties of standard solid and fluid materials are taken from the COMSOL Multiphysics material database unless otherwise specified.

The following assumptions and settings are adapted to develop the COMSOL model:

- The released rays are polychromatic with a bandwidth (300–2500 nm) with the number of rays specified as 10 000, and they are set to be unpolarized with intensity 1000 W/m^2^, with the fraction of direct sunlight ∼90% and diffuse ∼10%.
- Real and imaginary parts of the refractive index of the materials are specified through the default Medium Properties defined for all domains.
- A default Material Discontinuity boundary condition is generated for all boundaries to account for the reflection and refraction between different materials.
- To accurately model Fresnel lens concentration, solar rays are released as light cones with a disk angle of 4.65 mrad, accounting for the sun's angular divergence caused by its finite size [54, 55].
- An air gap between the SPV and the lens is considered for accurate estimation of the reflection at the exit interface of the SPV and the front interface of the lens.
- The reflection from the lens to the SPV is computed from the Material Discontinuity node, a default feature applied to all boundaries.
- As the thermal absorber is placed in a vacuum, the heat loss by convection and conduction is negligibly small.
- Heat Flux nodes are assigned for the exterior boundaries of the STPV module, lens, glass, and the compartment, flux type is chosen to be convective heat flux.
- Surface‐to‐Ambient radiation boundary conditions are added for each of the SPV module, lens, glass, and the compartment. The surface emissivity is defined for each associated boundary, and the ambient temperature is set as 25°C.
- Blackbody/Graybody is selected in the initial values boundary condition.
- Custom diffuse surfaces are provided for different surfaces with assigning the associated emissivity and defining the ambient temperature (*T*amb = 25°C), and layer opacity is generated at each diffuse surface to determine whether the surface is transparent or opaque to the emitted radiation from the surface.
- Radiative thermal exchanges within collector channels are neglected [56].
- The flow is considered steady, fully laminar, and incompressible [57].
- No‐slip condition is imposed on the wall to state that the fluid velocity at the channels’ walls is zero [58].

#### 4.2.1 Ray Optics

All ray tracing phenomena are simulated by employing Snell's law and Fresnel equations. The ray trajectory can be computed by solving six coupled first‐order ordinary differential equations for the components of the 3D ray vector *k*, the position vector *q*, and angular frequency *ω*.

(5) $$\frac{dk}{dt}=-\frac{\partial \omega }{\partial q}\;q,k=x,y,z$$

(6) $$\frac{dq}{dt}=-\frac{\partial \omega }{\partial k}\;q,k=x,y,z$$

#### 4.2.2 Heat Transfer and Fluid Dynamics

The heat transfer mechanism is considered as a conjugate heat transfer of conduction and convection in the fluid domain inside the channels, and it is solved by the following equation:

(7) $$\rho C_{p}u\cdot \nabla T+\nabla \cdot \left(-k\nabla T\right)=Q$$

The fluid dynamics inside the channels are governed by the Navier–Stokes Equations (8), representing the conservation of momentum, associated with the continuity Equation (9), which represents the conservation of mass. The equations are given at steady state [58].

(8) $$\rho \left(u.\nabla \right)u=\nabla .\left(-P\;I\right)+\nabla .\left(\mu \left(\nabla u+{\left(\nabla u\right)}^{T}\right)-\frac{2}{3}\mu \left(\nabla \cdot u\right)\;I\right)+F_{V}$$

(9) $$\nabla .\;\left(\rho u\right)=0$$

Radiative heat exchange is simulated using the Surface‐to‐Surface Radiation interface in the Radiation Heat Transfer Module of COMSOL Multiphysics. This interface is coupled to the Heat Transfer in Solids and Fluids interface through the Heat Transfer with Surface‐to‐Surface Radiation multiphysics coupling. The model calculates the irradiation incident on each participating surface and its radiosity, defined as the sum of emitted and reflected radiation. The radiosities of all participating surfaces are solved simultaneously, thereby accounting for repeated emission, interception, reflection, and re‐emission between the SPV module and Fresnel lens, as well as among the CST absorber, glazing, and enclosure walls.

In the vacuum chamber, radiative heat transfer is the dominant mechanism. The radiative heat flux is determined by both incoming and emitted radiation, with the latter represented by the radiosity *J*, which accounts for emitted and reflected radiation. For opaque, diffuse‐grey surfaces, the blackbody emissive power *e*_b_(*T*) is given by the Stefan–Boltzmann law.

(10) $$e_{b}\;\left(T\right)=\sigma T^{4}$$

The total incident irradiation *G* comprises ambient irradiation *G*_amb_, external irradiation *G*_ext_ coming from solar irradiance heat flux, and mutual irradiation *G*_m_ originating from other surfaces. The radiosity equation, derived from these relations, is implemented in COMSOL, with the net radiative heat flux incorporated into the heat transfer equation at boundaries.

(11) $$J=\varepsilon e_{b}\left(T\right)+\left(1-\varepsilon \right)\left(G_{\mathrm{amb}}+G_{\mathrm{ext}}+G_{m}\right)$$

(12) $$q_{r,\mathrm{net}}=\varepsilon \left(G-e_{b}\left(T\right)\right)$$

Heat source boundaries for SPV and CST are defined as general sources, with each boundary heat source *Q*_b_ set based on deposited ray power from ray tracing simulations. The SPV values are manually assigned, while CST values are directly linked to the GOP interface's sub‐node to account for the solar flux gradient from the Fresnel lens.

Heat loss from exterior surfaces, governed by Equations (13)–(15) [58, 59], occurs primarily through convection and radiation:

(13) $$q_{\mathrm{loss}}=q_{\mathrm{cv}}+q_{\mathrm{rad}}$$

Convective heat exchange with the surroundings *q*_cv_​ is modeled using a convective heat transfer coefficient h_cv,wind_​, dependent on wind speed υ_wind_. Various estimation methods exist [60, 61], with an intermediate expression (Equation 16) adopted for υ_wind_ < 5 m/s [42]. Convective heat flux follows Equation (15) for SPV outer surfaces, the Fresnel lens, and glazing, while for thermally insulated compartment walls, it is determined using Equation (17).

(14) $$q_{\mathrm{cv}}=h_{\mathrm{cv},\mathrm{wind}}\;\left(T-T_{\mathrm{amb}}\right)$$

(15) $$h_{\mathrm{cv},\mathrm{wind}}=4.5+2.9\upsilon_{\mathrm{wind}}$$

(16) $$q_{\mathrm{cv},\mathrm{ins}}=U_{ins}\;\left(T-T_{\mathrm{amb}}\right)$$

where *U*_ins_ is the heat transfer coefficient between the thermal compartment outer walls and the environment (through the insulation), calculated as follows:

(17) $$U_{\mathrm{ins}}=\frac{1}{\frac{\delta_{\mathrm{ins}}}{k_{\mathrm{ins}}}+\frac{1}{h_{\mathrm{cv},\mathrm{wind}}}}\;$$

where δ_ins_ and *k*_ins_ are the thickness and thermal conductivity of the insulation respectively.

The surface‐to‐ambient radiation boundary condition is used to model the heat radiation to the ambient, calculated from the following equation:

(18) $$q_{\mathrm{rad}}=\varepsilon \sigma \left(T^{4}-T_{\mathrm{amb}}^{4}\right)$$

where *ɛ* is the emissivity of each respective diffuse surface, and *σ* is the Stefan–Boltzmann constant.

The validation model is configured to reproduce the laboratory‐scale experiment as closely as possible, using the same materials and boundary conditions as the test setup. By contrast, the later full‐system simulations use parameters representative of a practical SPV‐CST collector design intended for high‐temperature operation. In addition, the temperature variation within the CST channels is explicitly resolved by the conjugated heat‐transfer and fluid‐dynamics model, while the electrical output of the SPV module is evaluated from its simulated operating temperature using the measured temperature coefficient.

## Author Contributions

G. H. and N. A. A developed the concept and research methodology. N. A. A. (lead) and P. A. developed the model. N. A. A. simulated and analyzed the performance of the device. N. A. A. (lead), Y. T., B. S. R., U. W. P. and G. H. analyzed data. N. A. A. (lead) and A. R. fabricated the prototype. D. R., B. A. N. and U. W. P. contributed to the perovskite solar cells. N. A. A. (lead) and G. H. conducted the validation experiments. All authors contributed to writing and revising the manuscript. G. H. directed the project.

## Conflicts of Interest

The authors declare no conflicts of interest.

## Supporting information




**Supporting File**: advs78362‐sup‐0001‐SuppMat.pdf.

## Data Availability

The data and numerical model that support the findings of this study are available from the corresponding author upon reasonable request.

## References

[1] IEA S. H. C ., Solar Heat Worldwide (2025), https://www.iea‐shc.org/solar‐heat‐worldwide‐2025.

[2] Fraunhofer ISE Photovoltaics Report, (2025), https://www.ise.fraunhofer.de/content/dam/ise/de/documents/publications/studies/Photovoltaics‐Report.pdf.

[3] D. Das , P. Kalita , and O. Roy , “Flat Plate Hybrid Photovoltaic‐ thermal (PV/T) System: A Review on Design and Development,” Renewable and Sustainable Energy Reviews 84 (2018): 111–130.

[4] A. Mellor , D. A. Alvarez , I. Guarracino , et al., “Roadmap for the Next‐Generation of Hybrid Photovoltaic‐Thermal Solar Energy Collectors,” Solar Energy 174 (2018): 386–398.

[5] G. Huang , K. Wang , and C. N. Markides , “Efficiency Limits of Concentrating Spectral‐Splitting Hybrid Photovoltaic‐Thermal (PV‐T) Solar Collectors and Systems,” Light: Science & Applications 10 (2021): 1–14. 10.1038/s41377-021-00465-1 PMC7862645 33542174

[6] F. Crisostomo , “Spectral Splitting Strategy and Optical Model for the Development of a Concentrating Hybrid PV/T Collector,” Applied Energy 141 (2015): 238–246.

[7] Z. Wu , J. Wang , L. Yin , et al., “Boosting Total Conversion Efficiency of Hybrid Photovoltaic–Thermal via a Spectral Splitter/Absorber Based on Lossy Periodic Structured Media,” Solar RRL 6, no. 8 (2022): 2200283.

[8] Z. Wu , J. Wang , Y. Liu , et al., “A Review of Spectral Controlling for Renewable Energy Harvesting and Conserving,” Materials Today Physics 18 (2021): 100388.

[9] X. Han , L. Tu , and Y. Sun , “A Spectrally Splitting Concentrating PV/T System Using Combined Absorption Optical Filter and Linear Fresnel Reflector Concentrator,” Solar Energy 223 (2021): 168–181.

[10] H. Liang , F. Wang , L. Yang , Z. Cheng , Y. Shuai , and H. Tan , “Progress in Full Spectrum Solar Energy Utilisation by Spectral Beam Splitting Hybrid PV/T System,” Renewable and Sustainable Energy Reviews 141 (2021): 110785.

[11] R. A. Taylor , T. Otanicar , and G. Rosengarten , “Nanofluid‐Based Optical Filter Optimisation for PV/T Systems,” Science & Applications 1 (2012): e34–e34.

[12] S. Guo , H. Cao , and J. Sun , “Proposal of a Parabolic‐Trough‐Type Spectral Splitting Concentrating Photovoltaic‐Photothermal (SSCPVT) System for Combined Power and Heat Generation,” Renewable Energy 242 (2025): 122483.

[13] B. Lehmann and G. Huang , “Hybrid Solar Collectors Based on Bragg‐Mirror Spectral Splitting for Simultaneous Heat and Electricity Generation,” Solar Energy 306 (2026): 114288.

[14] C. Pandey , M. Wu , A. Oyeniran , et al., “Numerical Study of a Parabolic‐Trough CPV‐T Collector With Spectral‐Splitting Liquid Filters,” Frontiers in Energy 19, no. 6 (2025): 949–968.

[15] B. Lehmann , B. S. Richards , and G. Huang , “Spectral‐Splitting Photovoltaic‐Thermal Solar Collectors Using Selective Hybrid Liquid‐Solid Optical Filters,” Advanced Optical Materials 14, no. 10 (2026): 02756.

[16] S. S. Chougule , B. A. Gaikwad , A. Inbaoli , A. Ajayan , S. K. Saha , and P. Estellé , “Hybrid Au‐ZnO/(Water‐Ethylene Glycol)‐Nanofluid Based PV/T Spectral Beam Splitter for Efficient Photo‐Harvesting,” Energy 331 (2025): 137055.

[17] H. Liang , F. Wang , L. Yang , Z. Cheng , Y. Shuai , and H. Tan , “Progress in Full Spectrum Solar Energy Utilization by Spectral Beam Splitting Hybrid PV/T System,” Renewable and Sustainable Energy Reviews 141 (2021): 110785.

[18] G. Huang , S. R. Curt , K. Wang , and C. N. Markides , “Challenges and Opportunities for Nanomaterials in Spectral Splitting for High‐Performance Hybrid Solar Photovoltaic‐thermal Applications: A Review,” Nano Materials Science 2 (2020): 183–203.

[19] D. S. Codd , M. D. Escarra , B. Riggs , et al., “Solar Cogeneration of Electricity With High‐Temperature Process Heat,” Cell Reports Physical Science 1 (2020): 100135.

[20] H. Liang , R. Su , W. Huang , et al., “A Novel Spectral Beam Splitting Photovoltaic/Thermal Hybrid System Based on Semi‐Transparent Solar Cell With Serrated Groove Structure for Co‐Generation of Electricity and High‐Grade Thermal Energy,” Energy conversion and management 252 (2022): 115049.

[21] G. Huang , P. H. Arya , D. B. Ritzer , et al., “Hybrid Perovskite‐Photovoltaic and Solar‐Thermal Harvesting,” Advanced Science 12, no. 42 (2025): 09692. 10.1002/advs.202509692 PMC12622551 40990045

[22] G. Huang and C. N. Markides , “Spectral‐splitting Hybrid PV‐Thermal (PV‐T) Solar Collectors Employing Semi‐Transparent Solar Cells as Optical Filters,” Energy Conversation and Management 248 (2021): 114776.

[23] J. Wen , H. Hu , C. Chen , et al., “Present Status of and Future Opportunities for All‐Perovskite Tandem Photovoltaics,” Nature Energy 10 (2025): 681–696.

[24] H. Zhu , S. Teale , M. N. Lintangpradipto , et al., “Long‐term Operating Stability in Perovskite Photovoltaics,” Nature Reviews Materials 8, no. 9 (2023): 569–586.

[25] F. Laufer , M. Götz , and U. W. Paetzold , “Deep Learning for Augmented Process Monitoring of Scalable Perovskite Thin‐Film Fabrication,” Energy & Environmental Science 18, no. 4 (2025): 1767–1782. 10.1039/d4ee03445g PMC11734647 39830789

[26] European Commission, Joint Research Centre , Photovoltaic Geographical Information System (PVGIS), accessed February 08 2024, https://re.jrc.ec.europa.eu/pvg_tools/en/.

[27] S. Perini , X. Tonnellier , P. King , and C. Sansom , “Theoretical and Experimental Analysis of an Innovative Dual‐axis Tracking Linear Fresnel Lenses Concentrated Solar Thermal Collector,” Solar Energy 153 (2017): 679–690.

[28] M. Gupta , A. K. Dubey , V. Kumar , and D. S. Mehta , “Experimental Study of Combined Transparent Solar Panel and Large Fresnel Lens Concentrator Based Hybrid PV/Thermal Sunlight Harvesting System,” Energy for Sustainable Development 63 (2021): 33–40.

[29] Y. Gao , L. Tang , H. Zhou , Y. Zhang , Z. Tan , and Q. Li , “Using Perovskite Solar Cells With Tunable Bandgaps for Beam‐Splitting Photovoltaic‐Thermal System,” International Journal of Green Energy 22, no. 6 (2025): 1052–1062.

[30] N. G. Park , “Perovskite Solar Cells: An Emerging Photovoltaic Technology,” Materials Today 18 (2015): 65–72.

[31] S. Bai , Z. Wu , X. Wu , et al., “High‐performance Planar Heterojunction Perovskite Solar Cells: Preserving Long Charge Carrier Diffusion Lengths and Interfacial Engineering,” Nano Research 7 (2014): 1749–1758.

[32] Y. Liu , L. Zhang , M. Wang , et al., “Bandgap‐tunable Double‐Perovskite Thin Films by Solution Processing,” Materials Today 28 (2019): 25–30.

[33] G. Huang , P. H. Arya , D. B. Ritzer , et al., “Hybrid Perovskite‐Photovoltaic and Solar‐Thermal Harvesting,” Advanced Science 12 (2025): 09692. 10.1002/advs.202509692 PMC12622551 40990045

[34] M. Schultes , T. Helder , E. Ahlswede , et al., “Sputtered Transparent Electrodes (IO: H and IZO) With Low Parasitic Near‐Infrared Absorption for Perovskite–Cu (In, Ga) Se_2_ Tandem Solar Cells,” ACS Applied Energy Materials 2, no. 11 (2019): 7823–7831.

[35] S. K. Jung , K. Park , D. K. Lee , J. H. Lee , H. Ahn , and J. W. Lee , “Effects of MgF_2_ Anti‐reflection Coating on Optical Losses in Metal Halide Perovskite Solar Cells,” Nanotechnology 35, no. 13 (2024): 135401. 10.1088/1361-6528/ad1647 38100835

[36] A. Lorusso , S. Masi , C. Triolo , et al., “A Rational Approach to Improve the Overall Performances of Semitransparent Perovskite Solar Cells by Electrode Optical Management,” ACS Energy Letters 9, no. 4 (2024): 1923–1931.

[37] Y. T. Yang , Q. Y. Cai , F. X. Li , et al., “Fabrication of Antireflection Coatings From Visible to Mid‐wave Infrared via Quantitative Control of the Refractive Index,” Optics Express 34, no. 3 (2026): 3676–3690. 10.1364/OE.581836 41715430

[38] H. A. Zondag , D. W. de Vries , W. G. J. van Helden , R. J. C. van Zolingen , and A. A. van Steenhoven , “The Yield of Different Combined PV‐Thermal Collector Designs,” Solar Energy 74, no. 3 (2003): 253–269, 10.1016/S0038-092X(03)00121-X.

[39] P. G. Charalambous , G. G. Maidment , S. A. Kalogirou , and K. Yiakoumetti , “Photovoltaic Thermal (PV/T) Collectors: A Review,” Applied Thermal Engineering 27 (2007): 275–286.

[40] A. Hassanzadeh , L. Jiang , and R. Winston , “Coupled Optical‐thermal Modeling, Design and Experimental Testing of a Novel Medium‐temperature Solar Thermal Collector With Pentagon Absorber,” Solar Energy 173 (2018): 1248–1261, 10.1016/j.solener.2018.08.022.

[41] C. K. Jeon , J. S. Lee , H. Chung , J. H. Kim , and J. P. Park , “A Study on Insulation Characteristics of Glass Wool and Mineral Wool Coated With a Polysiloxane Agent,” Advances in Materials Science and Engineering 2017 (2017): 3938965.

[42] M. Herrando , C. N. Markides , and K. Hellgardt , “A UK‐Based Assessment of Hybrid PV and Solar‐Thermal Systems for Domestic Heating and Power: System Performance,” Applied Energy 122 (2014): 288–309, 10.1016/j.apenergy.2014.01.061.

[43] Y. Ma , Y. Luo , H. Xu , R. Du , and Y. Wang , “Review on Air and Water Thermal Energy Storage of Buildings With Phase Change Materials,” Experimental and Computational Multiphase Flow 3, no. 2 (2021): 77–99.

[44] S. D. H. Naqvi , K. Son , W. Jung , et al., “Mitigating Intrinsic Interfacial Degradation in Semi‐Transparent Perovskite Solar Cells for High Efficiency and Long‐Term Stability,” Advanced Energy Materials 13, no. 47 (2023): 2302147, 10.1002/aenm.202302147.

[45] M. Nakamura , C. C. Lin , C. Nishiyama , K. Tada , T. Bessho , and H. Segawa , “Semi‐Transparent Perovskite Solar Cells for Four‐Terminal Perovskite/CIGS Tandem Solar Cells,” ACS Applied Energy Materials 5, no. 7 (2022): 8103–8111, 10.1021/acsaem.2c00620.

[46] National Laboratory of the Rockies , “Best Research‐Cell Efficiency Chart.” accessed April 17, 2026, https://www.nlr.gov/pv/cell‐efficiency.

[47] M. H. Miah , M. B. Rahman , M. Nur‐E‐Alam , et al., “Key Degradation Mechanisms of Perovskite Solar Cells and Strategies for Enhanced Stability: Issues and Prospects,” RSC advances 15, no. 1 (2025): 628–654, 10.1039/D4RA07942F. PMC11705851 39777157

[48] M. U. Ali , H. Mo , Y. Li , and A. B. Djurišić , “Outdoor Stability Testing of Perovskite Solar Cells: Necessary Step Toward Real‐Life Applications,” APL Energy 1, no. 2 (2023), 10.1063/5.0155845.

[49] S. W. Lee , S. Kim , S. Bae , et al., “UV Degradation and Recovery of Perovskite Solar Cells,” Scientific reports 6, no. 1 (2016): 38150, 10.1038/srep38150. PMC5133559 27909338

[50] B. P. Kore , M. Jamshidi , and J. M. Gardner , “The Impact of Moisture on the Stability and Degradation of Perovskites in Solar Cells,” Materials Advances 5, no. 6 (2024): 2200–2217, 10.1039/D3MA00828B.

[51] W. Chi and S. K. Banerjee , “Stability Improvement of Perovskite Solar Cells by Compositional and Interfacial Engineering,” Chemistry of Materials 33, no. 5 (2021): 1540–1570, 10.1021/acs.chemmater.0c04931.

[52] R. A. Afre and D. Pugliese , “Perovskite Solar Cells: A Review of the Latest Advances in Materials, Fabrication Techniques, and Stability Enhancement Strategies,” Micromachines 15, no. 2 (2024): 192, 10.3390/mi15020192. PMC10890723 38398920

[53] GCL , World's First Commercial Gigawatt‐scale Perovskite Production Base Laid Foundation GCL Embarks on a New Era of Global Perovskite Commercial Operations, accessed September 14 2026, https://www.gcl‐power.com/en/about/newdetail/5759.html.

[54] R. Stieglitz and W. Platzer , “Concentrating Collectors,” Solar Thermal Energy Systems: Fundamentals, Technology, Applications (2024): 603–753, 10.1007/978-3-031-43173-9_7.

[55] N. O. Onubogu , K. K. Chong , C. W. Wong , T. K. Yew , and B. H. Lim , “Optical Characterization of Two‐Stage Non‐Imaging Solar Concentrator for Active Daylighting system,” Solar Energy 185 (2019): 24–33, 10.1016/j.solener.2019.04.028.

[56] C. Cristofari , G. Notton , and J. L. Canaletti , “Thermal Behavior of a Copolymer PV/Th Solar System in Low Flow Rate Conditions,” Solar Energy 83, no. 8 (2009): 1123–1138, 10.1016/j.solener.2009.01.008.

[57] A. Nahar , M. Hasanuzzaman , and N. A. Rahim , “Numerical and Experimental Investigation on the Performance of a Photovoltaic Thermal Collector With Parallel Plate Flow Channel Under Different Operating Conditions in Malaysia,” Solar Energy 144 (2017): 517–528, 10.1016/j.solener.2017.01.041.

[58] M. Herrando , A. Ramos , I. Zabalza , and C. N. Markides , “A Comprehensive Assessment of Alternative Absorber‐Exchanger Designs for Hybrid PVT‐water Collectors,” Applied Energy 235 (2019): 1583–1602, 10.1016/j.apenergy.2018.11.024.

[59] A. Mahapatra and P. P. Tripathy , “Experimental Investigation and Numerical Modeling of Heat Transfer During Solar Drying of Carrot Slices,” Heat and Mass Transfer 55, no. 5 (2019): 1287–1300, 10.1007/s00231-018-2492-2.

[60] G. Notton , C. Cristofari , M. Mattei , and P. Poggi , “Modelling of a Double‐Glass Photovoltaic Module Using Finite Differences,” Applied Thermal Engineering 25, no. 17‐18 (2005): 2854–2877, 10.1016/j.applthermaleng.2005.02.008.

[61] J. Antonanzas , A. del Amo , A. Martinez‐Gracia , A. A. Bayod‐Rujula , and F. Antonanzas‐Torres , “Towards the Optimization of Convective Losses in Photovoltaic–Thermal Panels,” Solar Energy 116 (2015): 323–336, 10.1016/j.solener.2015.04.013.

