# Supplementary material for: Hybrid Semi‐Transparent Perovskite‐Photovoltaics and Concentrated Solar‐Thermal Harvesting Collectors
Source: Adv Sci (Weinh). 2026 Oct 20:e78362. Online ahead of print. doi: 10.1002/advs.78362 (PMC13683880; doi:10.1002/advs.78362)
Supplement: Supplementary file 1 [file ADVS-9999-e78362-s001.pdf]

# Hybrid semi-transparent perovskite-photovoltaics and concentrated solar-thermal harvesting collectors


Nada. A. Alati[1], Parth H. Arya[1], Yu Tian[1], Ashok Reddy[1], David B. Ritzer[1, 2], Bahram Abdollahi Nejand[1, 2], Ulrich W. Paetzold[1,2], Bryce S. Richards[1, 2], Gan Huang[1*]

[1] Institute of Microstructure Technology, Karlsruhe Institute of Technology, Hermann-von-Helmholtz-Platz 1, 76344 Eggenstein-Leopoldshafen, Germany.

[2] Light Technology Institute, Karlsruhe Institute of Technology, Engesserstrasse 13, 76131 Karlsruhe, Germany.

*Corresponding author: gan.huang@kit.edu


## Supplementary figures

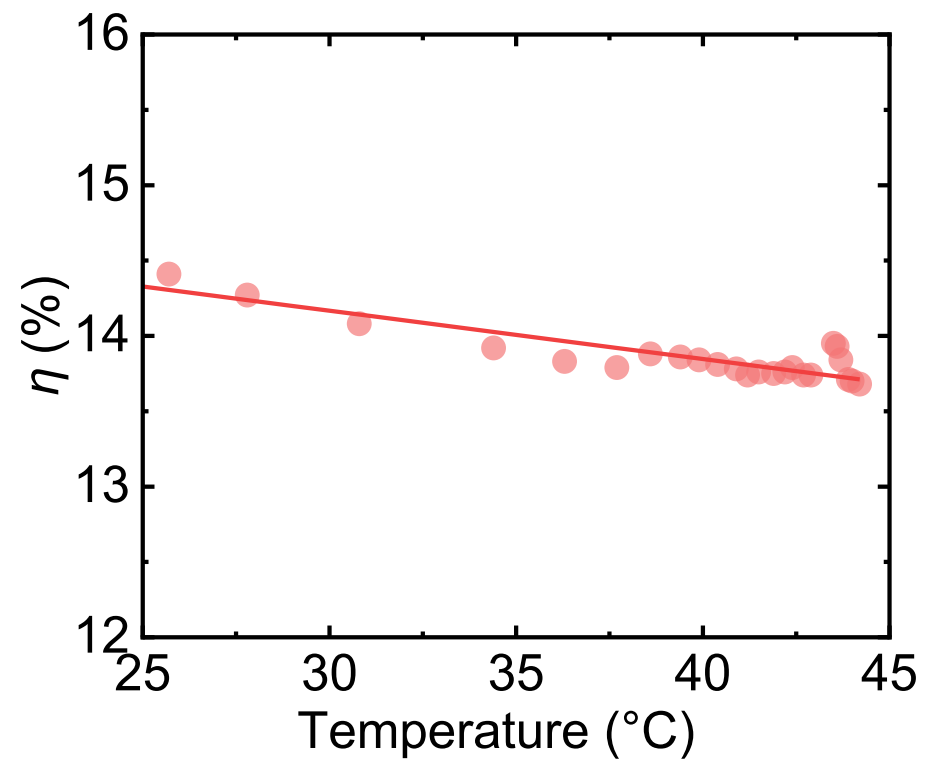


**Supplementary Figure 1.** Electrical efficiency of SPV as a function of operating temperature.

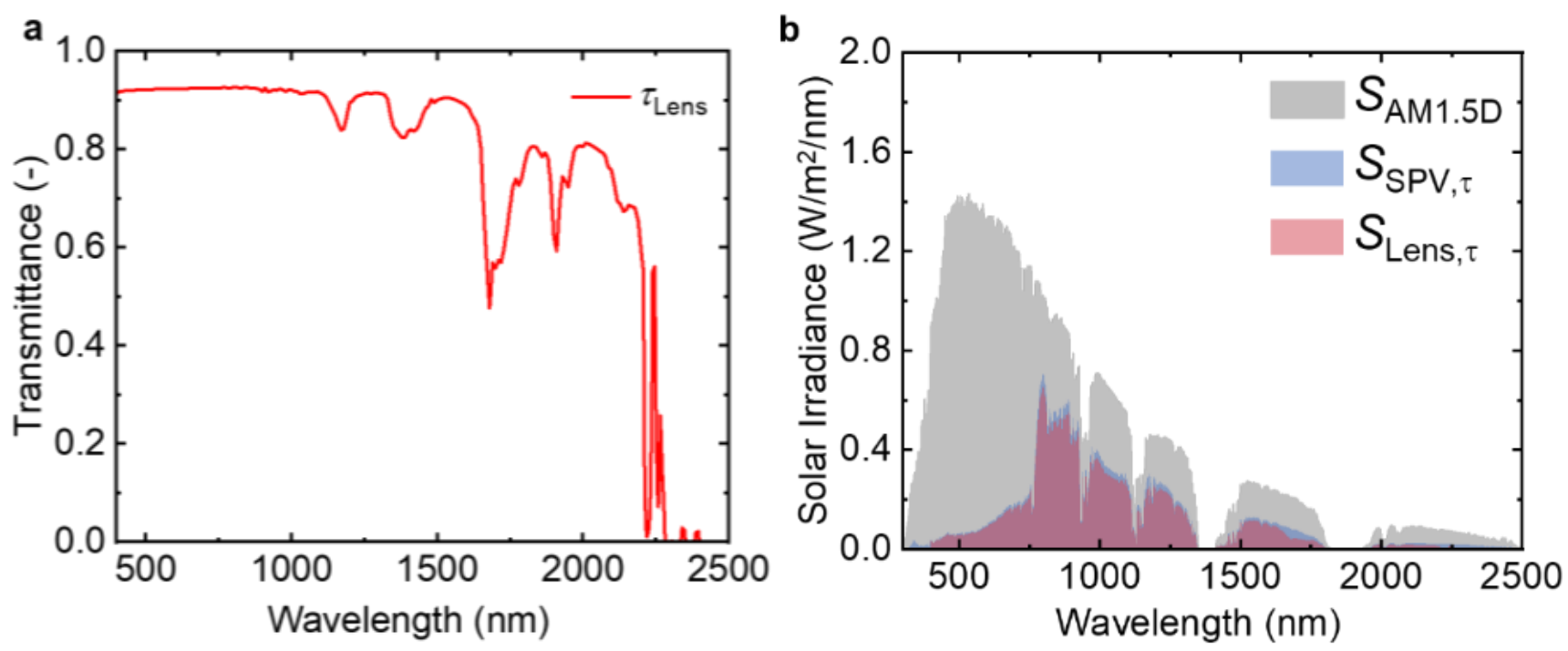


**Supplementary Figure 2** a) Transmittance of Fresnel lens. b) Comparison of direct solar irradiance through Perovskite versus spectrum transmitted through the lens.

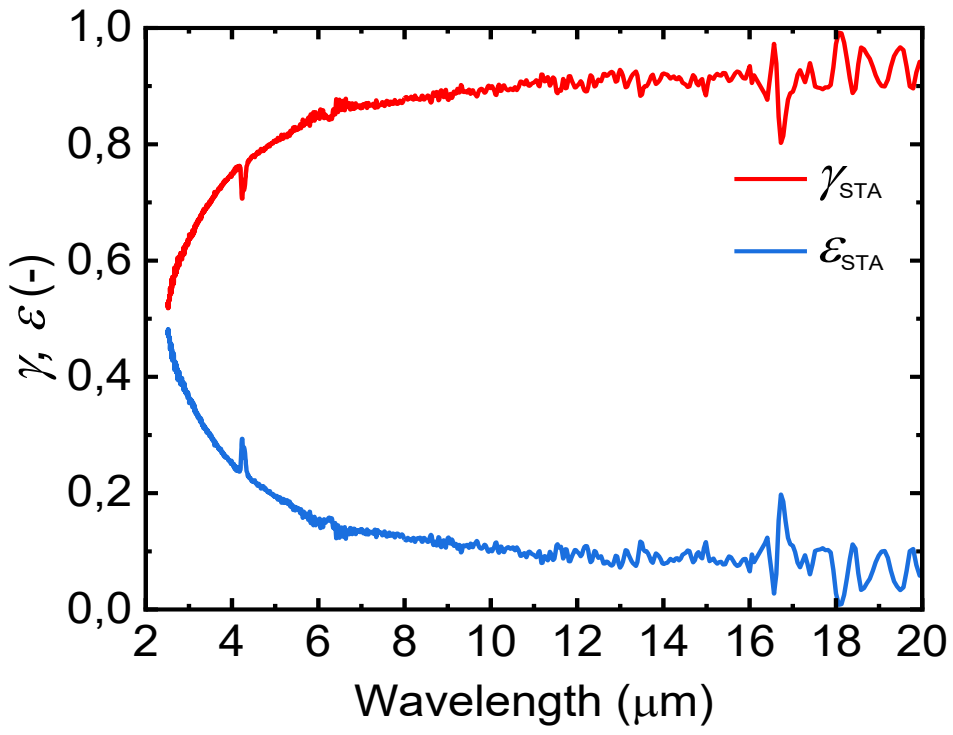


**Supplementary Figure 3**. Emissivity and reflectivity spectra of the low-emissivity solar thermal absorber in long wavelength bandwidth 2-20 μm.

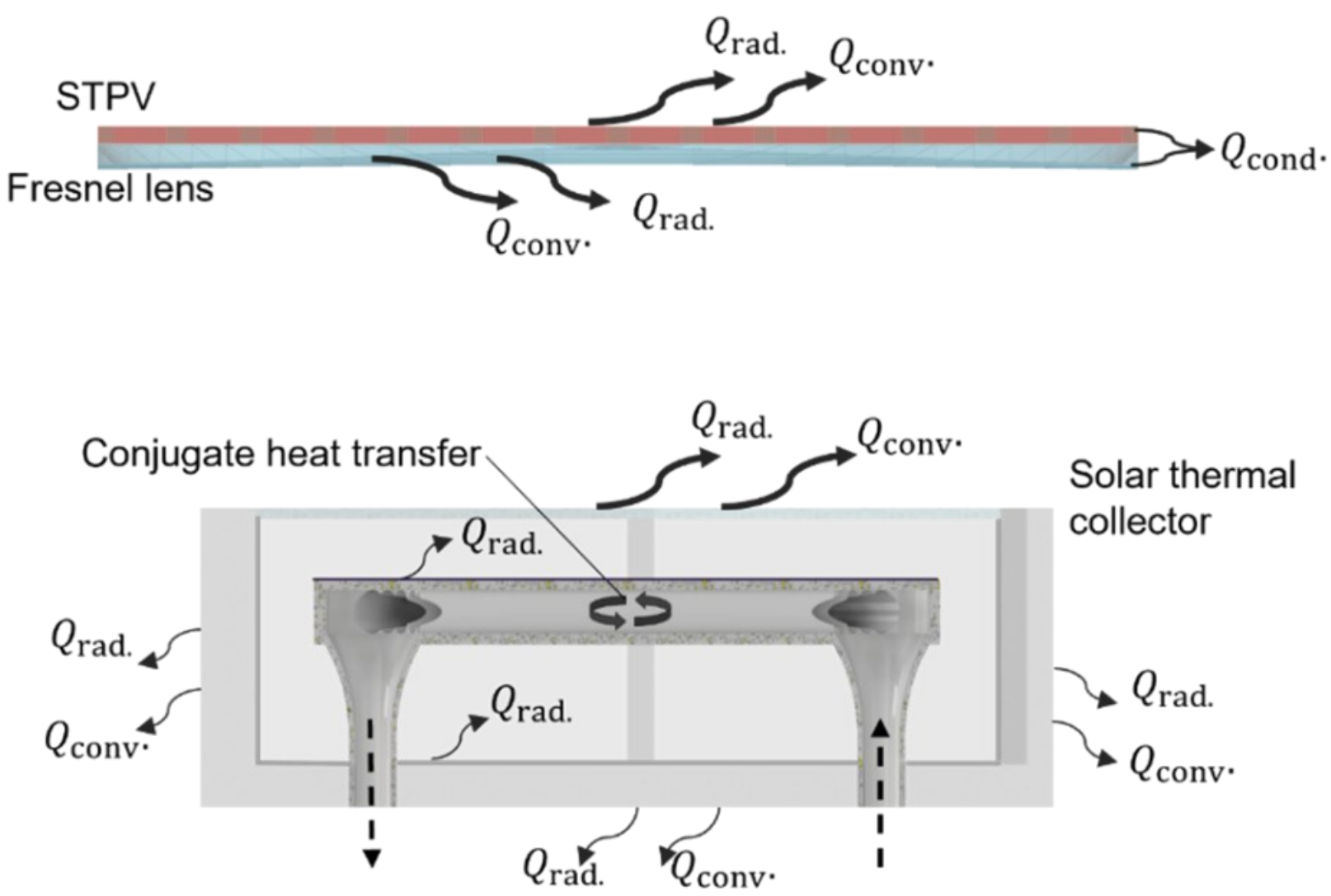


**Supplementary Figure 4. Schematic diagram illustrating the heat transfer mechanisms occurring in various domains and surfaces of the SPV/CST collector**. In all solid domains, heat conduction takes place where the heat is transported by means of vibrating particles. Inside the channels forced convection dominates the heat transfer in the flowing fluid. Heat convection occurs from the outer surfaces of all the components to the surrounding air. Heat is radiated from the emissive exterior walls to the ambient. In addition to radiative heat exchange takes place between the sides of the thermal absorber, the inner sides of the thermal compartment (low emissivity coating), and the glazing.

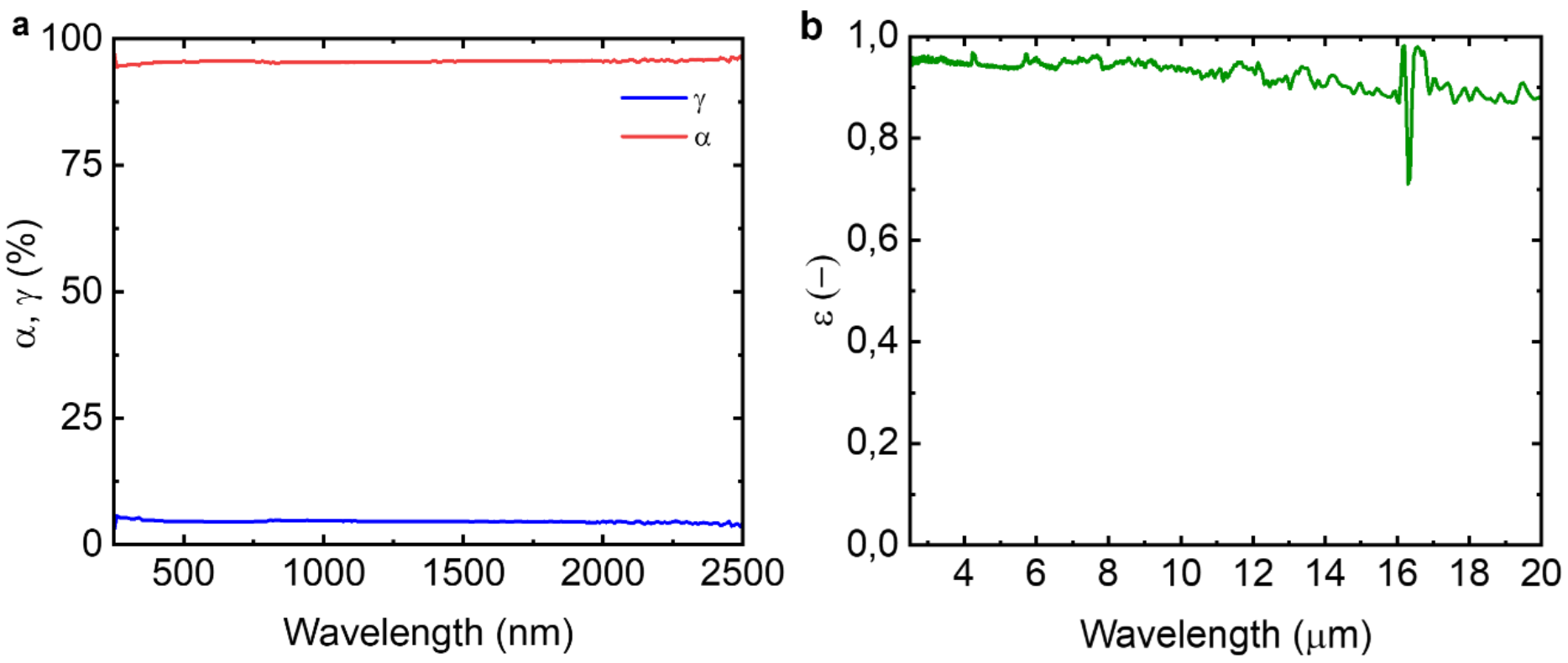


**Supplementary Figure 5. Optical properties of painted solar thermal absorber**. a) Absorptance and reflectance of the painted STA used for validation in the band width 250-2500 nm. b) The emissivity spectrum in the bandwidth 2-20 µm.

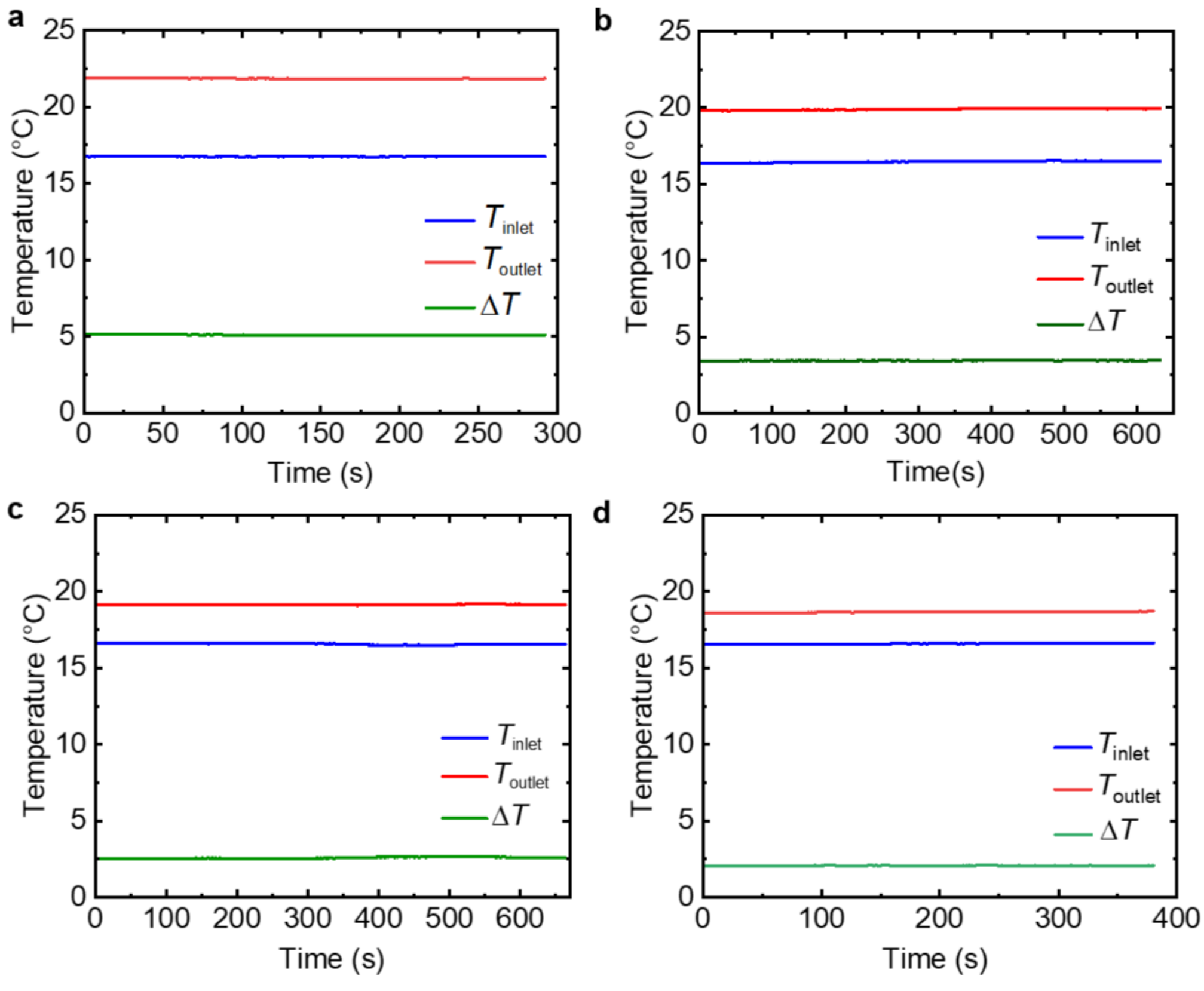


**Supplementary Figure 6. Experimental data of the inlet and outlet temperature during steady state situation for different mass flow rates.** a) 1.5 kg/h, b) 2.2 kg/h, c) 3.1 kg/h, and d) 3.8 kg/h.

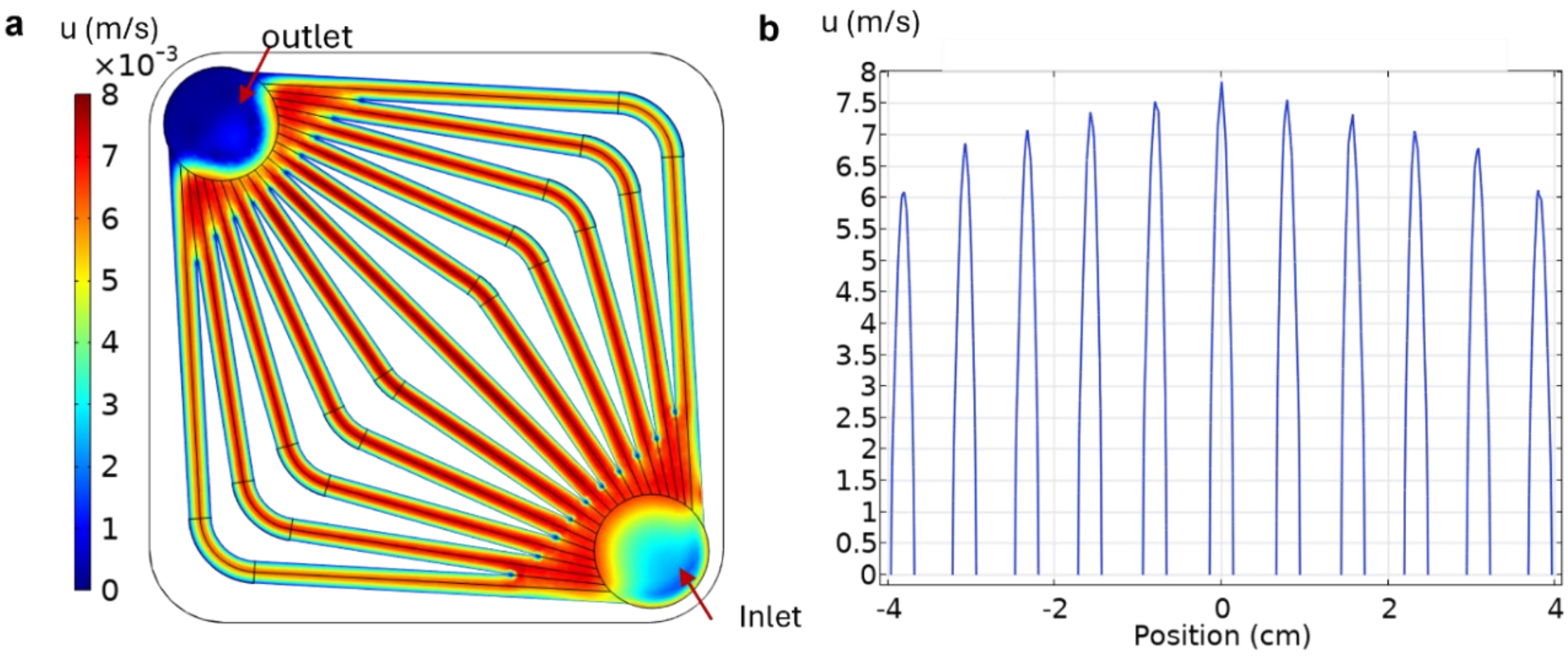


**Supplementary Figure 7. Fluid dynamics and velocity distribution in the STA.** (a) Velocity gradient within individual channels on a cross-sectional plane through the middle of the channels. The fluid velocity is highest at the channel center, where wall influence is minimal, and gradually decreases toward the walls due to viscosity effects and the no-slip condition. A region of negligible velocity is observed at the top corner of the outlet tube, where flow does not reach, forming a low-flow region or dead zone. (b) Velocity magnitude along a central cut line through the channels, exhibiting a parabolic velocity profile characteristic of laminar flow.

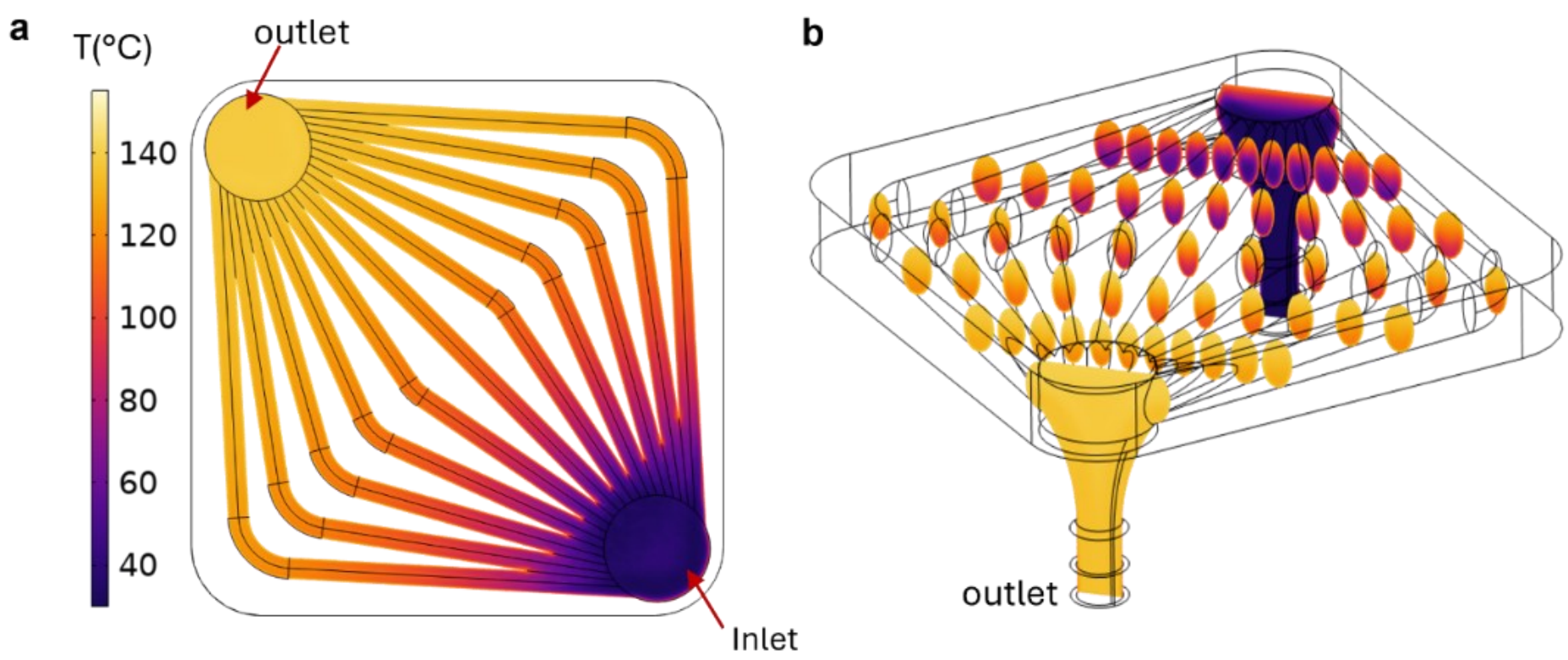


**Supplementary Figure 8. Temperature distribution in the fluid domain of the STA.** (a) Temperature distribution on a cross-sectional plane through the middle of the channels, illustrating the evolution of fluid temperature as it flows through the channels of the STA. (b) Temperature distribution of the heat transfer fluid at the inlet, channels and outlet, demonstrating a uniform temperature profile at the outlet. The results highlight the importance of achieving uniform thermal distribution for efficient heat transfer in practical applications.

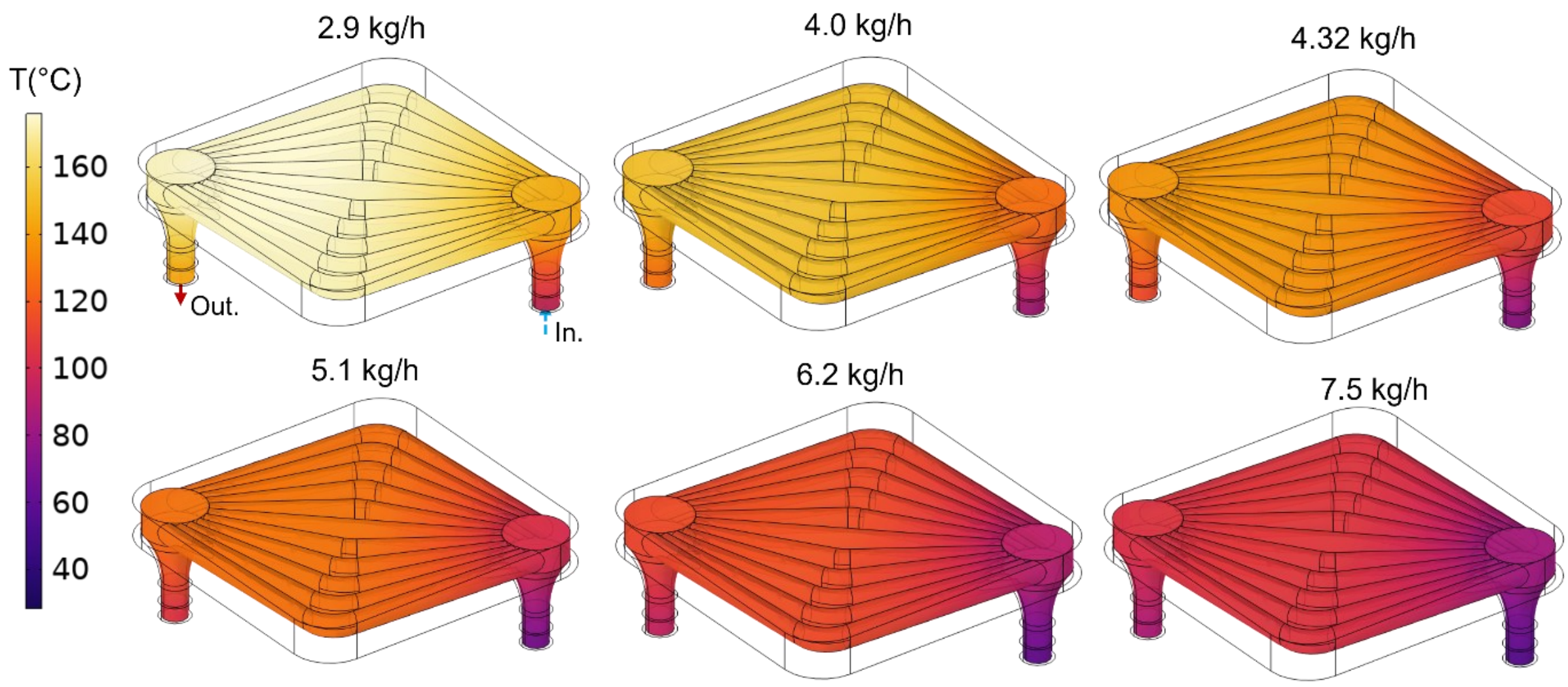


**Supplementary Figure 9.** The impact of varying mass flow rate on the temperature distribution of the heat transfer oil (HTO), as the flow rate increases the temperature of HTO decreases.

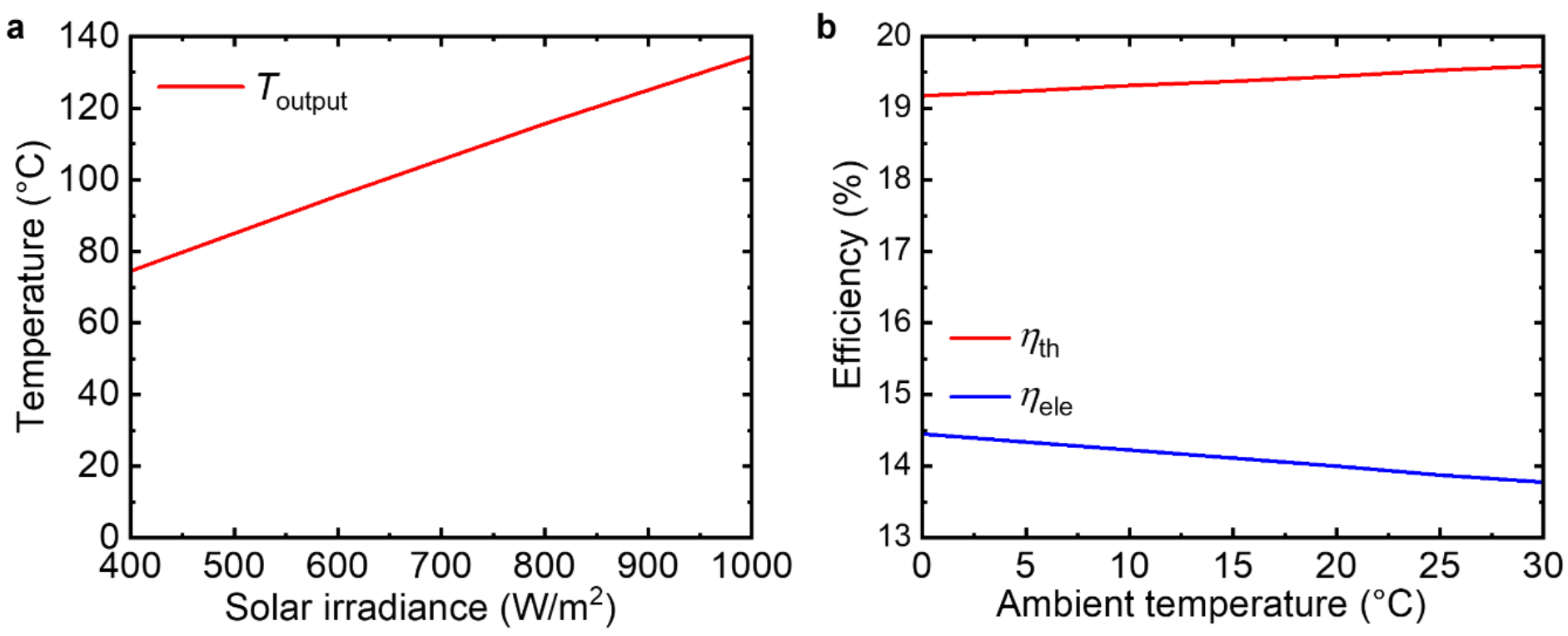


**Supplementary Figure 10. Impact of weather.** a) Thermal performance of the concentrating solar thermal collector under various solar irradiance, showing that thermal efficiency increases with higher solar irradiance. b) Variations in thermal and electrical efficiencies with ambient temperature. The thermal efficiency demonstrates a slight increase at higher ambient temperatures due to good insulation, while the electrical efficiency decreases at higher ambient temperatures.

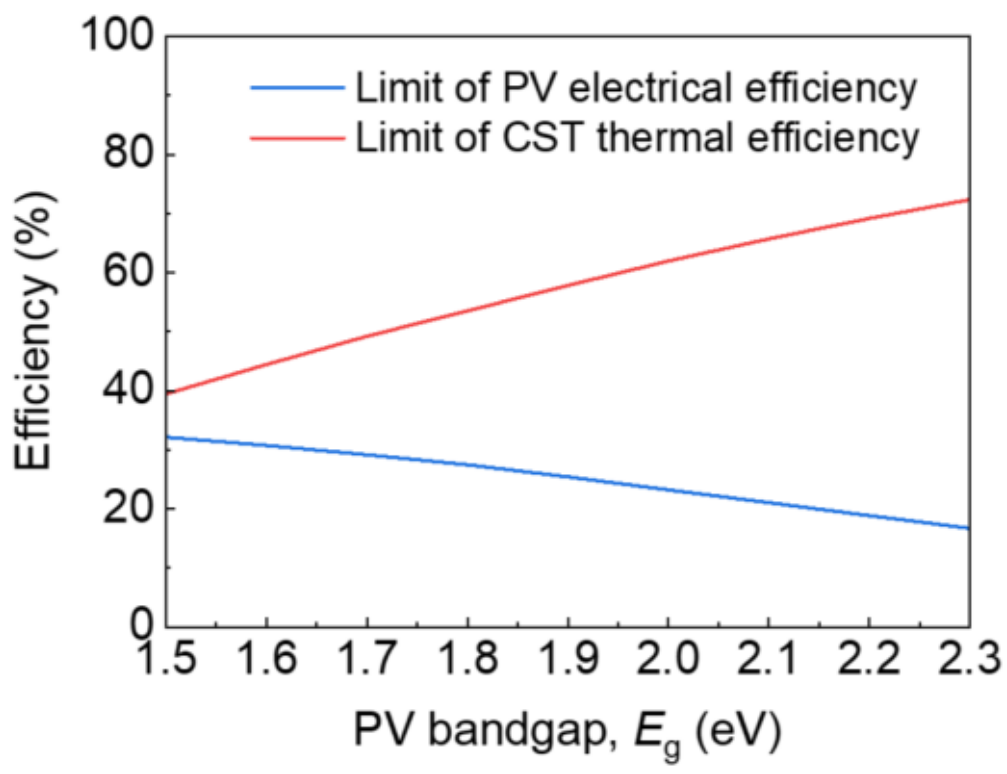


**Supplementary Figure 11. Theoretical influence of the perovskite bandgap on photovoltaic and thermal-energy harvesting.** Shockley-Queisser limit of photovoltaic electrical efficiency and the ideal upper limit of CST thermal efficiency as functions of the photovoltaic bandgap. The thermal limit is calculated from the fraction of

the AM1.5D solar spectrum at wavelengths longer than the corresponding bandgap absorption edge, assuming ideal conversion of the transmitted sub-bandgap solar energy into useful heat.

**Supplementary Table 1. Input parameters of the validation model**

| | Parameter | Value |
|---|---|---|
| $\alpha_{\mathrm{STA}}$ | absorptivity of thermal absorber | 0.96 |
| $\gamma_{\mathrm{STA}}$ | reflectivity of thermal absorber | 0.04 |
| $\varepsilon_{\mathrm{STA}}$ | emissivity of thermal absorber | 0.94 |
| $\varepsilon_{\mathrm{Al}}$ | emissivity of Alloy Aluminium | 0.24 |
| $\varepsilon_{\mathrm{PMMA}}$ | emissivity of PMMA | 0.90 |
| $\varepsilon_{\mathrm{wall}}$ | emissivity of the inner walls coating | 0.04 |
| $k_{\mathrm{Al}}$ | thermal conductivity of Alloy Aluminium | 130 W/m.K |
| $h_{\mathrm{cv}}$ | heat transfer coefficient | 5 W/m$^2$.K |

**Supplementary Table 2**. Summary of key performance of selected spectral-splitting PVT collectors

| References | Spectral-splitting optical filters | Electrical efficiency | Thermal efficiency | Maximum heat temperature | Diffuse light tolerance |
|---|---|---|---|---|---|
| Liang et al. [1] | Nanofilms | 18.8% | 4.2% at 45 °C | ~45 °C | No |
| Lehmann et al. [2] | Nanofilms | 6.5% | 8.8% at 80 °C | 209.1 °C | No |
| Liang et al. [3] | Nanofluids | 13.1% | 10.9% | N/A | No |
| Otanicar et al. [4] | Nanofluids | 5% | 61% | 115 °C | No |
| Gupta et al. [5] | Semi-transparent cadmium telluride PV module | 3.4% | 63% at ~75 °C | ~75 °C | Yes for PV; no for thermal branch |
| This study | Semi-transparent perovskite PV module (SPV1/SPV2/SPV3) | 13.8%/ 21.3%/ 18.9% | 20.7%/ 19.2%/ 21.6% at 100 °C | >150 °C | Yes for PV; no for thermal branch |

**References:**

[1] Liang, H., Han, H., Wang, F., Cheng, Z., Lin, B., Pan, Y., & Tan, J. (2019). Experimental investigation on spectral splitting of photovoltaic/thermal hybrid system with two-axis sun tracking based on SiO2/TiO2 interference thin film. Energy Conversion and Management, 188, 230-240.

[2] Lehmann, B., & Huang, G. (2026). Hybrid solar collectors based on Bragg-mirror spectral splitting for simultaneous heat and electricity generation. Solar Energy, 306, 114288.

[3] Huaxu, L., Fuqiang, W., Dong, Z., Ziming, C., Chuanxin, Z., Bo, L., & Huijin, X. (2020). Experimental investigation of cost-effective ZnO nanofluid based spectral splitting CPV/T system. Energy, 194, 116913.

[4] Otanicar, T., Dale, J., Orosz, M., Brekke, N., DeJarnette, D., Tunkara, E., ... & Harikumar, P. (2018). Experimental evaluation of a prototype hybrid CPV/T system utilizing a nanoparticle fluid absorber at elevated temperatures. Applied energy, 228, 1531-1539.

[5] Gupta, M., Dubey, A. K., Kumar, V., & Mehta, D. S. (2021). Experimental study of combined transparent solar panel and large Fresnel lens concentrator based hybrid PV/thermal sunlight harvesting system. Energy for Sustainable Development, 63, 33-40.
